# Supplementary material for: Homeostasis at different backgrounds: The roles of overlayed feedback structures in vertebrate photoadaptation
Source: PLoS One. 2023 Apr 28;18(4):e0281490. doi: 10.1371/journal.pone.0281490 (PMC10146485; doi:10.1371/journal.pone.0281490)
Supplement: S1 Text — Applied step perturbations lead to slower resetting kinetics for increasing backgrounds. (ZIP) [file pone.0281490.s002.zip › S1 Text.pdf]

Homeostasis at different backgrounds: The roles of overlayed feedback structures in vertebrate photoadaptation

Jonas V. Grini, Melissa Nygård, Peter Ruoff*

Department of Chemistry, Bioscience, and Environmental Engineering, University of Stavanger, Stavanger, Norway

Supporting Information S1 Text

Response kinetics of controllers m3 and m5

Controller m3

As for m1 the m3 controller has a direct activation of the compensatory flux $j_3{=}k_3{\cdot}E$ by E. A on its side inhibits the synthesis of E (with inhibition constant k_8).

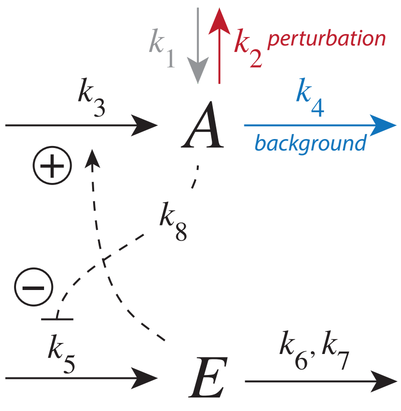

Fig S1. Controller motif m3 with integral control implemented as a zero-order Michaelis-Menten (MM) type degradation of E. As for the other controllers k_2 represents a perturbation, while k_4 is a background reaction. k_6 and k_7 are MM parameters analogous to V_{max} and K_M, respectively. The grayed-out rate constant k_1 is set to zero.

The rate equations of m3 are:

$$\dot{A} = k_1 - (k_2 + k_4){\cdot}A + k_3{\cdot}E \tag{S1}$$

$$\dot{E} = \frac{k_5 k_8}{k_8{+}A} - \frac{k_6{\cdot}E}{k_7{+}E} \tag{S2}$$

When applying zero-order conditions in the removal of E, i.e. $E/(k_7{+}E){\approx}1$ the steady state condition for E determines the controller's set-point, i.e.

$$\dot{E}{=}0 \;\Rightarrow\; \frac{k_5 k_8}{k_8{+}A_{ss}} = k_6 \;\Rightarrow\; A_{ss}{=}A_{set}{=}k_8\left(\frac{k_5}{k_6} - 1\right) \tag{S3}$$

A_{set} is set to 3.0 by choosing k_5=31.0, k_6=1.0, and k_8=0.1 with the same inhibition constant value as for the m2 controller.

Fig S2a shows that the m3's response kinetics are very similar to that of the m1 controller with increasingly delayed A resetting kinetics as backgrounds k_4 increase. We note that the m3 controller is somewhat slower in the A resetting in comparison with m1, possibly due to the inhibition of E synthesis by A (compare insets in Fig S2 and Fig 3a. Despite the differences in the A resetting between controllers 1 and 3, the steady state of E in Fig S2b is practically identical to that of Fig 3b.

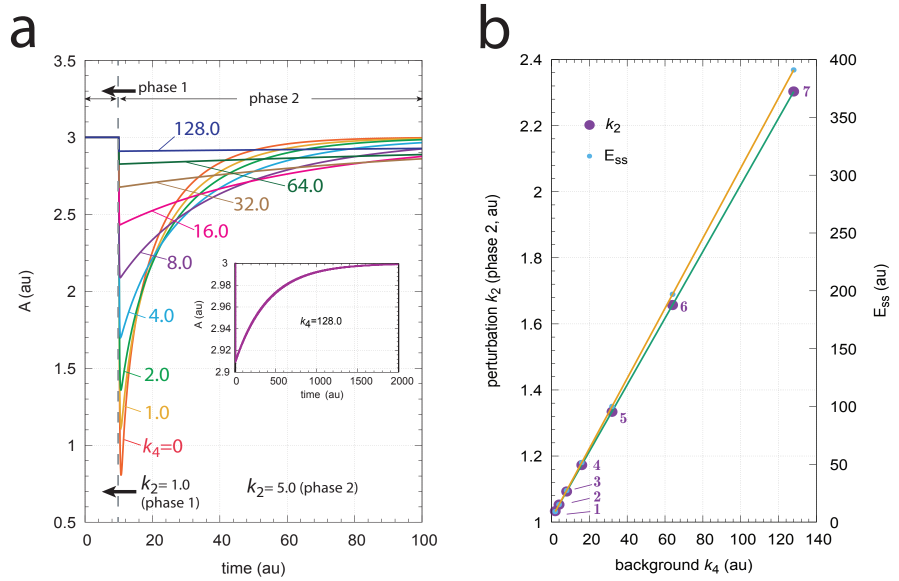

Fig S2. Response kinetics and Weber's law in the m3 controller (Fig S1). (a) Step-wise increase of k_2 from 1.0 to 5.0 at time t=10 at different and constant background perturbations k_4 (0-128.0, phases 1 and 2). As for the m1 controller we observe a successive decrease in the excursion of A (ΔA_{max}) with the A resetting kinetics slowed down (see inset). Rate constants: k_1=0.0, k_2=1.0 (phase 1), k_2=5.0 (phase 2), k_3=1.0, k_4 variable, k_5=31.0, k_6=1.0, $k_7=1\times10^{-6}$, k_8=0.1. Initial concentrations: A_0=3.0, E_0=3.0 (k_4=0); A_0=3.0, E_0=6.0 (k_4=1); A_0=3.0, E_0=9.0 (k_4=2); A_0=3.0, E_0=15.0 (k_4=4); A_0=3.0, E_0=27.0 (k_4=8); A_0=3.0, E_0=51.0 (k_4=16); A_0=3.0, E_0=99.0 (k_4=32); A_0=3.0, E_0=195.0 (k_4=64); A_0=3.0, E_0=387.0 (k_4=128). The inset shows the entire adaptation response when k_4=128.0 (b) Weber's law: the "perception" E_{ss} is a linear function of the background perturbation k_4 when ΔA_{max}=0.03. Rate constants and initial concentrations as in (a), except that k_2 in phase 2 has the following values: **1**, $k_2 = 1.0332$ ($k_4 = 2$); **2**, $k_2 = 1.0529$ ($k_4 = 4$); **3**, $k_2 = 1.0926$ ($k_4 = 8$); **4**, $k_2 = 1.1728$ ($k_4 = 16$); **5**, $k_2 = 1.3340$ ($k_4 = 32$); **6**, $k_2 = 1.6570$ ($k_4 = 64$); **7**, $k_2 = 2.3033$ ($k_4 = 128$).

Controller m5

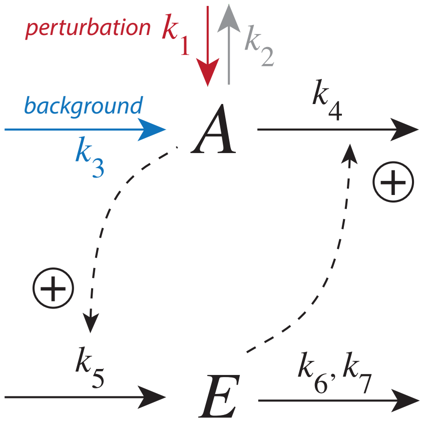

Fig S3. Controller motif m5 with integral control implemented as a zero-order Michaelis-Menten (MM) type degradation of E. Like the m1 controller the feedback loop in the m5 scheme is based on two activations, but shows outflow control, i.e. the controller compensates primarily inflow perturbations with k_3 as a background pertubation and k_1 undergoing a step-wise change.

The rate equations are:

$$\dot{A} = k_1 + k_3 - (k_2 + k_4{\cdot}E){\cdot}A \tag{S4}$$

$$\dot{E} = k_5{\cdot}A - \frac{k_6{\cdot}E}{k_7{+}E} \tag{S5}$$

Applying zero-order conditions in the removal of E, i.e. $E/(k_7{+}E){\approx}1$ the steady state condition for E determines the controller's set-point

$$\dot{E}{=}0 \;\;\Rightarrow\;\; k_5{\cdot}A_{ss} = k_6 \;\;\Rightarrow\;\; A_{ss}{=}A_{set}{=}\frac{k_6}{k_5} \tag{S6}$$

Fig S4a shows the m5's response kinetics, which are very similar to that of the m7 controller with increasingly delayed A resetting kinetics as backgrounds k_3 increase. Fig S4b shows that the m5 controller follows Weber's law when a just noticeable threshold of ΔA_{max}=0.03 (1% of A_{set}'s value) is considered. Panel c shows ΔA_{max} as a function of background k_3 using three different step-perturbations in k_1. As for the other controllers ΔA_{max} increases with increasing k_1 steps. Panel c shows t_{max} as a function of background k_3, but t_{max} is little affected by the magnitude of the k_1 step in comparison with ΔA_{max} (panel c).

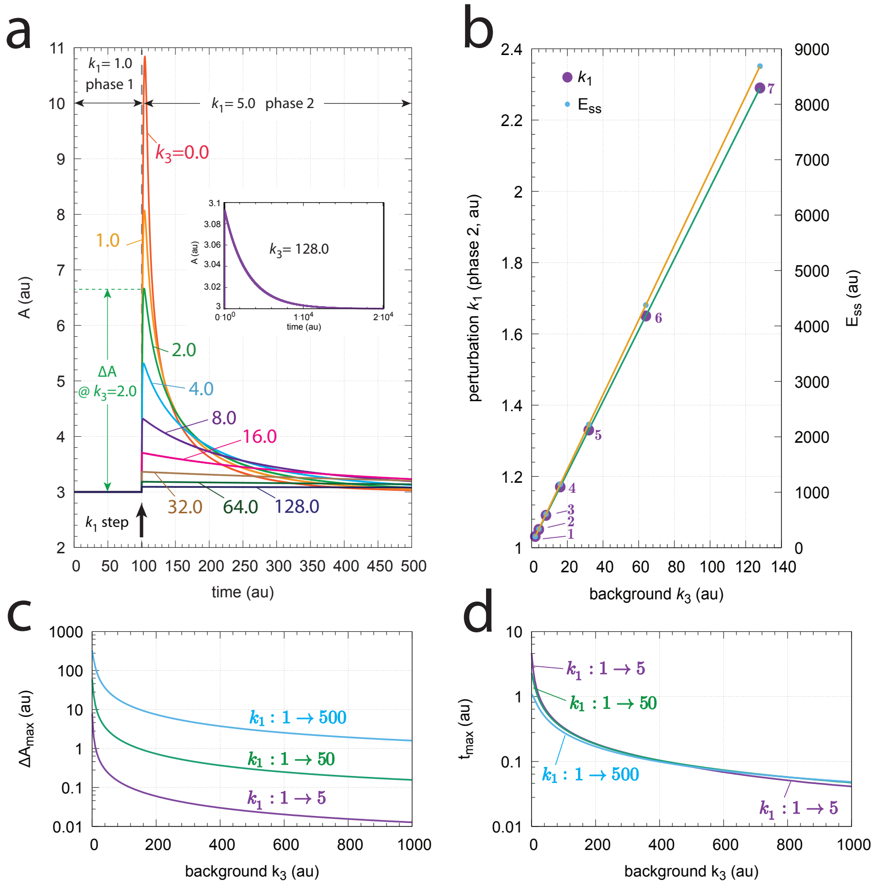

Fig S4. Response kinetics and Weber's law of the m5 controller (Fig S3). (a) Step-wise increase of k_1 from 1.0 to 5.0 at time t=100 at different and constant background perturbations k_3 (0-128.0, phases 1 and 2). As for the m7 controller we observe a successive decrease in the excursion of A (ΔA_{max}) with the A resetting kinetics slowed down (see inset). Rate constants: k_1=1.0 (phase 1), k_1=5.0 (phase 2), k_2=0.0, k_3 variable, k_4=0.005, k_5=1.0, k_6=3.0, k_7=1×10^{-6}. Initial concentrations: A_0=3.0, E_0=66.667 (k_3=0); A_0=3.0, E_0=133.33 (k_3=1); A_0=3.0, E_0=200.0 (k_3=2); A_0=3.0, E_0=333.33 (k_3=4); A_0=3.0, E_0=600.0 (k_3=8); A_0=3.0, E_0=1133.33 (k_3=16); A_0=3.0, E_0=2200.0 (k_3=32); A_0=3.0, E_0=4333.33 (k_3=64); A_0=3.0, E_0=8600.0 (k_3=128). The inset shows the entire adaptation response when k_3=128.0. (b) Weber's law: the "perception" E_{ss} is a linear function of the background perturbation k_3 when ΔA_{max}=0.03. Rate constants and initial concentrations as in (a), except that k_1 in phase 2 has the following values: **1**, $k_1 = 1.0316$ ($k_3 = 2$); **2**, $k_1 = 1.0512$ ($k_3 = 4$); **3**, $k_1 = 1.0908$ ($k_3 = 8$); **4**, $k_1 = 1.1705$ ($k_3 = 16$); **5**, $k_1 = 1.3303$ ($k_3 = 32$); **6**, $k_1 = 1.6502$ ($k_3 = 64$); **7**, $k_1 = 2.2901$ ($k_3 = 128$). (c) ΔA_{max} as a function of background k_3 using three different step-perturbations in k_1. (d) t_{max} as a function of background k_3 with the same step perturbations in k_1 as in panel c.
