# Supplementary material for: Homeostasis at different backgrounds: The roles of overlayed feedback structures in vertebrate photoadaptation
Source: PLoS One. 2023 Apr 28;18(4):e0281490. doi: 10.1371/journal.pone.0281490 (PMC10146485; doi:10.1371/journal.pone.0281490)
Supplement: S2 Text — Applied step perturbations lead to faster resetting kinetics for increasing backgrounds. (ZIP) [file pone.0281490.s003.zip › S2 Text.pdf]

Homeostasis at different backgrounds: The roles of overlayed feedback structures in vertebrate photoadaptation

Jonas V. Grini, Melissa Nygård, Peter Ruoff*

Department of Chemistry, Bioscience, and Environmental Engineering, University of Stavanger, Stavanger, Norway

Supporting Information S2 Text

Response kinetics of controllers m4 and m6.

Controller m4

In the m4 controller (Fig S1) the compensatory flux is, as for m2, based on derepression.

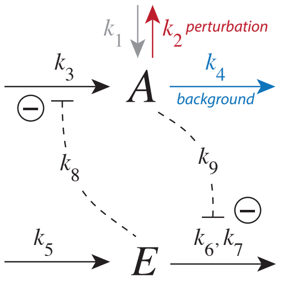

Fig S1. Controller motif m4. Integral control is implemented as a zero-order Michaelis-Menten (MM) type degradation of E. k_2 represents a perturbation and k_4 is a background reaction. k_6 and k_7 are MM parameters analogous to V_{max} and K_M, respectively. The grayed-out rate constant k_1 is set to zero.

The rate equations are:

$$\dot{A} = k_1 - k_2{\cdot}A - k_4{\cdot}A + \frac{k_3 k_8}{k_8{+}E} \tag{S1}$$

$$\dot{E} = k_5 - \left(\frac{k_6{\cdot}E}{k_7{+}E}\right)\cdot\left(\frac{k_9}{k_9{+}A}\right) \tag{S2}$$

The set-point A_{set} of the m4 controller is determined by finding the steady-state value of A from the condition $\dot{E}$=0 when $E/(k_7{+}E)\approx 1$, i.e.,

$$\dot{E}{=}0 \;\;\Rightarrow\;\; \frac{k_6 k_9}{k_9{+}A_{ss}} = k_5 \;\;\Rightarrow\;\; A_{ss}{=}A_{set}{=}k_9\left(\frac{k_6}{k_5} - 1\right) \tag{S3}$$

For k_5=1, k_6=31, and inhibition constant k_9=0.1 A_{set}=3.0.

Due to the derepression kinetics of the compensatory flux $j_3{=}k_3k_8/(k_8{+}E)$, the m4 controller behaves very similar as m2, i.e. increasing backgrounds of k_4 lead to reduced ΔA_{max} values, and to a more rapid resetting of A to its set-point A_{set} (Fig S2a).

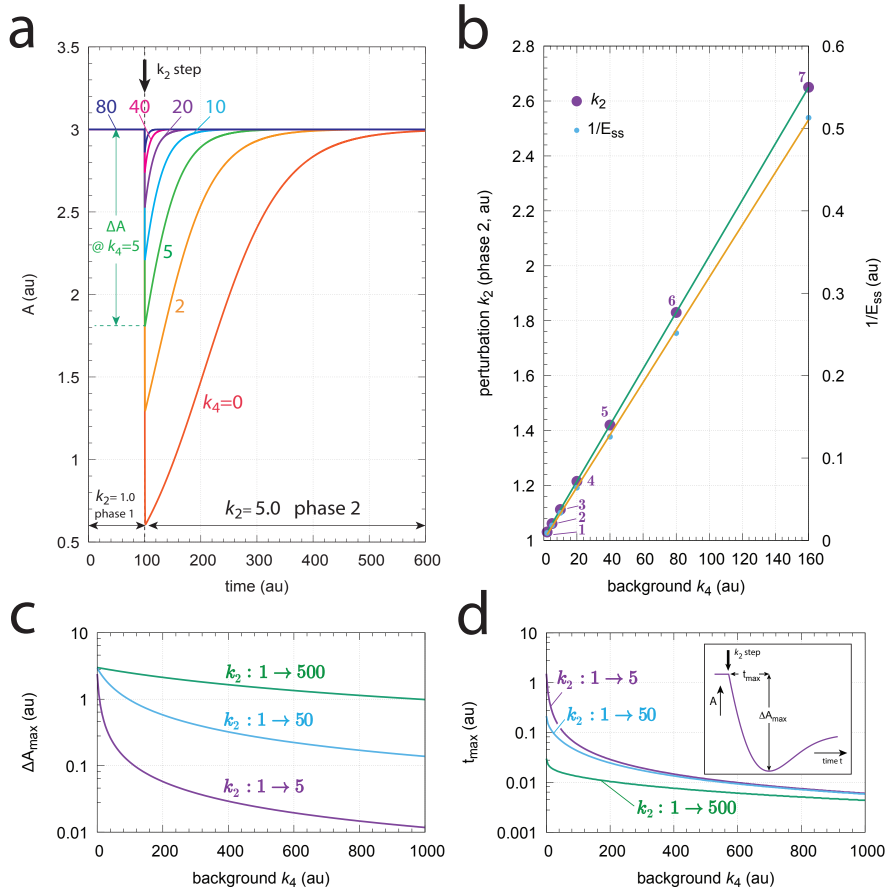

Fig S2. Response kinetics and Weber's law in the m4 controller (Fig S1). (a) Step-wise increase of k_2 from 1.0 to 5.0 at time t=100 at different and constant background perturbations k_4 (0-80). Note the successive decrease in the excursion of A (ΔA_{max}) and the more rapid A resetting kinetics at increased k_4 values. Rate constants: k_1=0.0, k_2=1.0 (phase 1), k_2=5.0 (phase 2), k_3=1×10^4, k_4 variable, k_5=1.0, k_6=31.0, k_7=1×10^{-6}, k_8=k_9=0.1. Initial concentrations: A_0=3.0, E_0=333.23 (k_4=0); A_0=3.0, E_0=111.01 (k_4=2); A_0=3.0, E_0=55.46 (k_4=5); A_0=3.0, E_0=30.20 (k_4=10); A_0=3.0, E_0=15.77 (k_4=20); A_0=3.0, E_0=8.03 (k_4=40); A_0=3.0, E_0=4.02 (k_4=80). (b) Weber's law related: the perturbation k_2 in phase 2 (left ordinate) is a linear function of the background perturbation k_4 when the "just noticable difference" ΔA_{max} is 0.03. Rate constants and initial concentrations as in (a), except that k_2 in phase 2 has the following values: **1**, $k_2 = 1.0308$ ($k_4 = 2$); **2**, $k_2 = 1.0615$ ($k_4 = 5$); **3**, $k_2 = 1.1127$ ($k_4 = 10$); **4**, $k_2 = 1.2152$ ($k_4 = 20$); **5**, $k_2 = 1.420$ ($k_4 = 40$); **6**, $k_2 = 1.830$ ($k_4 = 80$); **7**, $k_2 = 2.650$ ($k_4 = 160$). Right ordinate: $1/\mathrm{E_{ss}}$ is a linear function of the background perturbation k_4. (c) Maximum A-excursion ($\Delta\mathrm{A_{max}}$) as a function of k_2-step size and background k_4. (d) $\mathrm{t_{max}}$ (time of $\Delta\mathrm{A_{max}}$) after the step. See inset for definition of $\Delta\mathrm{A_{max}}$ and $\mathrm{t_{max}}$.

Fig S2b shows that for the m4 controller the "perception" $1/E_{ss}$ (right ordinate) becomes a linear function and resembles Weber's law for different background k_4's when the "just noticeable difference" in A is defined as 1% of A's set-point value (i.e., 0.03) (left ordinate). Fig S2c shows that the maximum excursion in A after the step decreases monotonically with increasing backgrounds k_4, but increases with increasing k_2 steps.

In panel (d) t_{max} is shown as a function of background k_4 and the k_2 step size. t_{max} decreases monotonically with increasing backgrounds k_4, but is somewhat dependent on the k_2 step size. The inset in panel (d) shows the definitions of ΔA_{max} and t_{max}.

Controller m6

In the controller loop m6 (Fig S3) the outflow-based compensatory flux $j_4 = k_4 k_8 \cdot A/(k8+E)$ is based on derepression. The perturbation k_1 is outlined in red and k_3 is the background (outlined in blue).

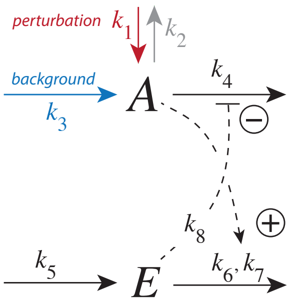

Fig S3. Controller motif m6. This is a derepression-based outflow controller with integral control implemented as a zero-order Michaelis-Menten (MM) type degradation of E. k_1 represents a perturbation and k_3 is a background reaction. k_6 and k_7 are MM parameters analogous to V_{max} and K_M, respectively. The grayed-out rate constant k_2 is set to zero.

The rate equations are:

$$\dot{A} = k_1 + k_3 - k_2 \cdot A - \left(\frac{k_4 \cdot k_8}{k_8 + E} \right) \cdot A \tag{S4}$$

$$\dot{E} = k_5 - \left(\frac{k_6 \cdot E}{k_7 + E} \right) \cdot A \tag{S5}$$

The set-point A_{set} is determined by finding the steady-state value of A from the condition $\dot{E}$=0 when $E/(k_7+E) \approx 1$, i.e.,

$$\dot{E}=0 \;\; \Rightarrow \;\; k_5 - k_6 \cdot A_{ss} = 0 \;\; \Rightarrow \;\; A_{ss} = A_{set} = \frac{k_5}{k_6} \tag{S6}$$

For k_5=6.0 and k_6=2.0 we have that A_{set}=3.0.

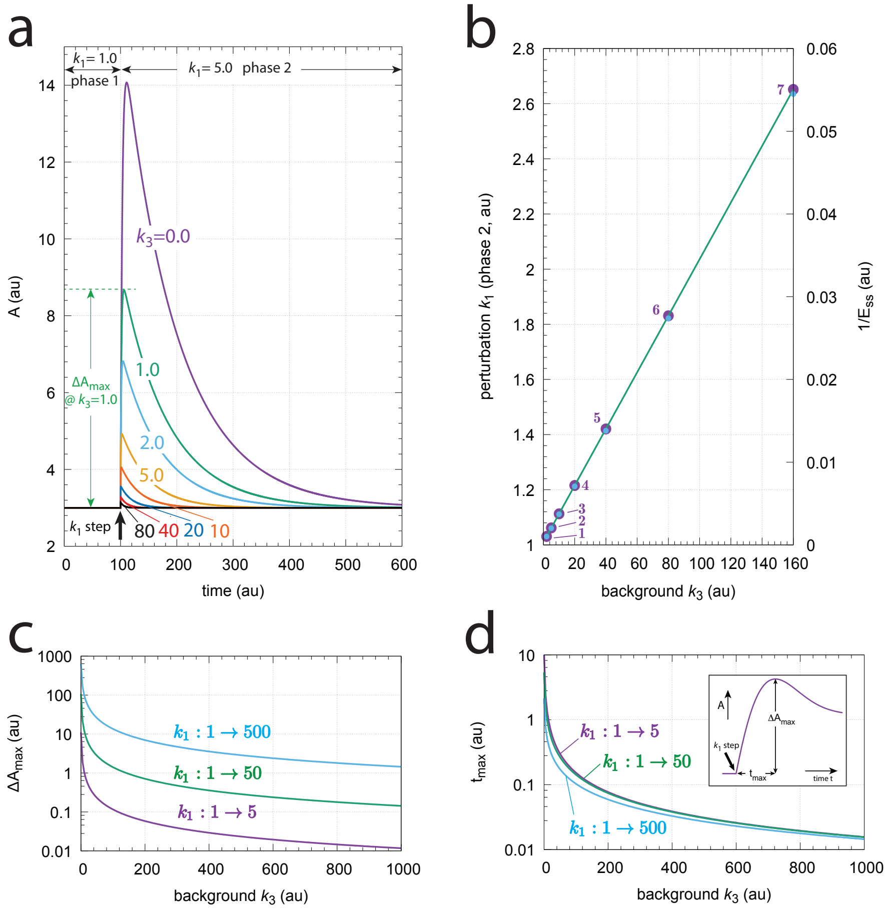

Fig S4. Response kinetics and Weber's law in the m6 controller (Fig S3). (a) Step-wise increase of k_1 from 1.0 to 5.0 at time t=100 at different and constant background perturbations k_3 (0-80). Note the successive decrease in the excursion of A (ΔA_{max}) and the more rapid A resetting kinetics at increased k_3 background values. Rate constants: k_1=1.0 (phase 1), k_1=5.0 (phase 2), k_2=0.0, k_3 variable, k_4=1×10^4, k_5=6.0, k_6=2.0, k_7=1×10^{-6}, k_8=0.1. Initial concentrations: A_0=3.0, E_0=2999.90 (k_3=0); A_0=3.0, E_0=1499.90 (k_3=1); A_0=3.0, E_0=999.90 (k_3=2); A_0=3.0, E_0=499.90 (k_3=5); A_0=3.0, E_0=272.63 (k_3=10); A_0=3.0, E_0=142.76 (k_3=20); A_0=3.0, E_0=73.07 (k_3=40); A_0=3.0, E_0=36.94 (k_3=80). (b) Weber's law related: the perturbation k_1 in phase 2 (left ordinate) is a linear function of the background perturbation k_3 when the "just noticable difference" ΔA_{max} is 0.03. Rate constants and initial concentrations as in (a), except that k_1 in phase 2 has the following values: **1**, $k_1 = 1.0308$ ($k_3 = 2$); **2**, $k_1 = 1.0616$ ($k_3 = 5$); **3**, $k_1 = 1.1128$ ($k_3 = 10$); **4**, $k_1 = 1.2154$ ($k_3 = 20$); **5**, $k_1 = 1.4205$ ($k_3 = 40$); **6**, $k_1 = 1.8308$ ($k_3 = 80$); **7**, $k_1 = 2.6515$ ($k_3 = 160$). Right ordinate: $1/\mathrm{E_{ss}}$ is a linear function of the background perturbation k_3. (c) Maximum A-excursion ($\Delta \mathrm{A_{max}}$) as a function of k_1-step size and background k_3. (d) $\mathrm{t_{max}}$ (time of $\Delta \mathrm{A_{max}}$) after the step. See inset for definition of $\Delta \mathrm{A_{max}}$ and $\mathrm{t_{max}}$.

Fig S4a shows, that due to the depression kinetics of the outflow control the relaxation time of the controller decreases with increasing background k_3. As for all the other controllers, ΔA_{max} decreases with increasing background. Fig S4b shows that $1/E_{ss}$ (right ordinate) is a linear function of background k_3 and resembles Weber's law with a "just noticeable difference" in A of 0.03) (left ordinate). Fig S4c shows that the

maximum excursion in A after the step decreases monotonically with increasing backgrounds k_3, but ΔA_{max} increases with increasing k_1 steps. In panel (d) $\mathrm{t_{max}}$ is shown as a function of k_3 and the k_1 step size. $\mathrm{t_{max}}$ decreases monotonically with increasing backgrounds k_3, but depends little on the k_1 step size. The inset in panel (d) shows the definitions of $\Delta \mathrm{A_{max}}$ and $\mathrm{t_{max}}$.
