# Supplementary material for: Homeostasis at different backgrounds: The roles of overlayed feedback structures in vertebrate photoadaptation
Source: PLoS One. 2023 Apr 28;18(4):e0281490. doi: 10.1371/journal.pone.0281490 (PMC10146485; doi:10.1371/journal.pone.0281490)
Supplement: S3 Text — The behavior is dynamically identical to that of m2 with zero-order kinetics. (ZIP) [file pone.0281490.s004.zip › S3 Text/S3 Text.pdf]

Homeostasis at different backgrounds: The roles of overlayed feedback structures in vertebrate photoadaptation

Jonas V. Grini, Melissa Nygård, Peter Ruoff*

Department of Chemistry, Bioscience, and Environmental Engineering, University of Stavanger, Stavanger, Norway

Supporting Information S3 Text

Response kinetics of controller m2 with antithetic integral control.

Fig S1 shows the scheme of the m2 feedback loop with antithetic integral control [1–4]

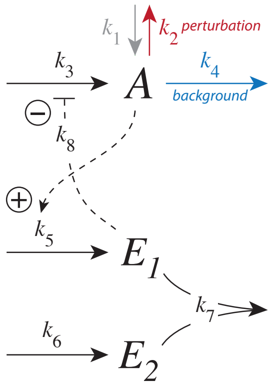

Fig S1. Controller motif m2 with antithetic integral control.

The rate equations are

$$\dot{A} = k_1 - k_2{\cdot}A - k_4{\cdot}A + \frac{k_3 k_8}{k_8{+}E_1} \tag{S1}$$

$$\dot{E}_1 = k_5{\cdot}A - k_7{\cdot}E_1{\cdot}E_2 \tag{S2}$$

$$\dot{E}_2 = k_6 - k_7{\cdot}E_1{\cdot}E_2 \tag{S3}$$

Making the steady state assumption for E_2, i.e. $\dot{E}_2 = 0$, we get that $k_6{=}k_7{\cdot}E_1{\cdot}E_2$ and that

$$\dot{E}_1 = k_5{\cdot}A - k_6 \tag{S4}$$

Eq S4 shows that the rate of E_1 becomes zero-order with respect to E_1, like the rate of E in Eq 12 becomes zero-order with respect to E when k_7 values are low with respect to E. Thus, E_1 and E have identical dynamical behaviors. This is shown in Fig S2 when a k_2 $1 \rightarrow 5$ step is applied in both m2 models with a background $k_4{=}0$.

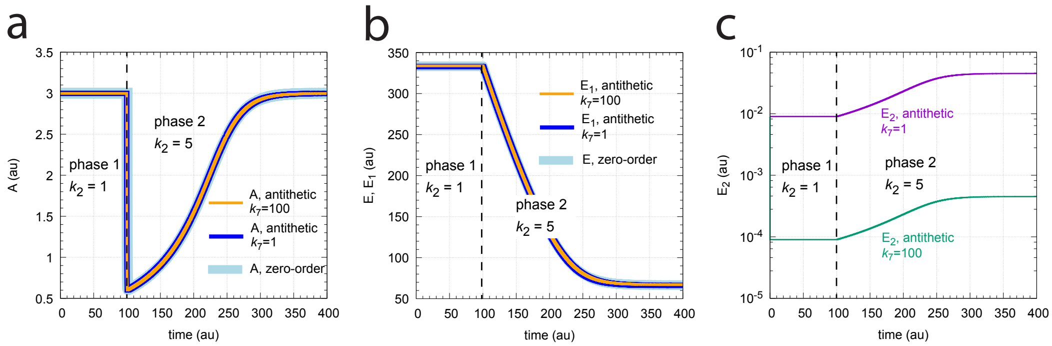

Fig S2. Comparison between m2 (Michaelis-Menten) zero-order controller (Eqs 10-11) and the m2 antithetic controller (Eqs 13-15). Panel a shows the dynamical behavior of the controlled variable A, which is independent of controller type and rate constant k_7. In panel b we see that the controller (manipulated) variables E, and E_1 behave identical, also here independent of controller type and rate constant k_7. Panel c shows that the concentration of the controller variable E_2 in the antithetic controller is dependent on k_7. In both controller models a k_2 1→5 step is applied at time t=100 with background k_4=0. Other rate constants, m2 (Michaelis-Menten) zero-order controller: k_1=0, $k_3=1\times10^4$, k_5=1.0, k_6=3.0, $k_7=1\times10^{-6}$, k_8=0.1. Other rate constants, m2 antithetic controller: k_1=0, $k_3=1\times10^4$, k_5=1.0, k_6=3.0, k_7=1 or 100, k_8=0.1. Initial concentrations, m2 (Michaelis-Menten) zero-order controller: A_0=3.0000, $E_0=3.3323\times10^2$. Initial concentrations, m2 antithetic controller (both when k_7=1 (thick blue line) or k_7=100 (thin orange line)): A_0=3.0000, $E_{1,0}=3.3323\times10^2$, $E_{2,0}=9.0027\times10^{-3}$.

We calculated ΔA_{max} and t_{max} for the m2 antithetic controller with rate constants described in Fig S2, which proved to be identical to those of the m2 zero-order controller (Eqs 10-11). Fig S3 shows the results.

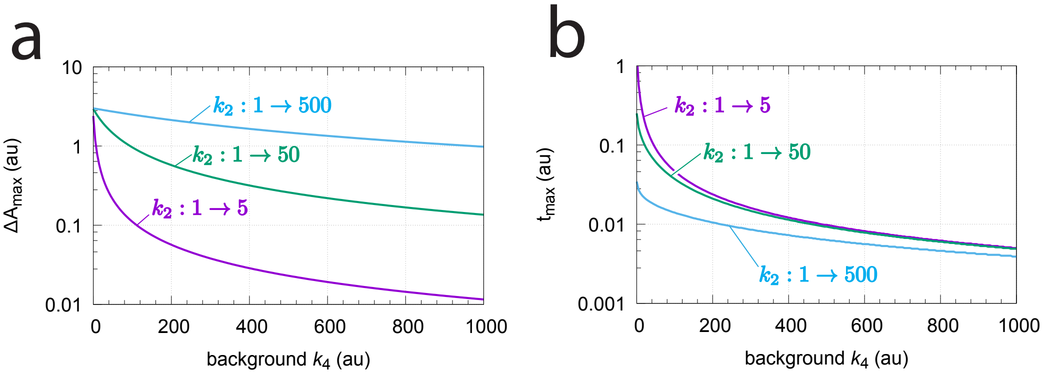

Fig S3. ΔA_{max} and t_{max} (Fig 2a) as a function of background k_4 for the m2 antithetic controller. Rate parameters and initial conditions as described in Fig S2 but k_4 starts at 0 and ends at 1000 with increments of 5. The numerical data are identical to that of the m2 controller described in Figs 8c and d.

References

1. Briat C, Gupta A, Khammash M. Antithetic integral feedback ensures robust perfect adaptation in noisy biomolecular networks. Cell Systems. 2016;2(1):15–26.

2. Briat C, Zechner C, Khammash M. Design of a synthetic integral feedback circuit: dynamic analysis and DNA implementation. ACS Synthetic Biology. 2016;5(10):1108–1116.

3. Aoki SK, Lillacci G, Gupta A, Baumschlager A, Schweingruber D, Khammash M. A universal biomolecular integral feedback controller for robust perfect adaptation. Nature. 2019;570(7762):533–537.

4. Waheed Q, Zhou H, Ruoff P. Kinetics and mechanisms of catalyzed dual-E (antithetic) controllers. PloS One. 2022;17(8):e0262371.
