# Supplementary material for: Homeostasis at different backgrounds: The roles of overlayed feedback structures in vertebrate photoadaptation
Source: PLoS One. 2023 Apr 28;18(4):e0281490. doi: 10.1371/journal.pone.0281490 (PMC10146485; doi:10.1371/journal.pone.0281490)
Supplement: S4 Text — A comparison how experimentally observed zero-order and first-order Ca leak kinetics affect photoadaptation in the model and when homeostatic breakdown occurs. (ZIP) [file pone.0281490.s005.zip › S4 Text.pdf]

Homeostasis at different backgrounds: The roles of overlayed feedback structures in vertebrate photoadaptation

Jonas V. Grini, Melissa Nygård, Peter Ruoff*

Department of Chemistry, Bioscience, and Environmental Engineering, University of Stavanger, Stavanger, Norway

Supporting Information S4 Text

Influence of Ca leak kinetics on photoadaptation

Experimental Ca leak kinetics

Ca leak kinetic data from the literature [1–4] show both first-order and zero-order kinetics with respect to store (endoplasmatic reticulum, ER) calcium. Fig S1a shows extracted data from the work by Camello et al. [3], which indicates close to zero-order kinetics with an approximately constant removal rate of Ca out of the ER. On the other hand, Fig S1b shows clean first-order kinetics with an exponentially decreasing removal rate of Ca out of the ER. We have chosen these two data sets, because in these two studies absolute calcium concentrations inside the ER were reported.

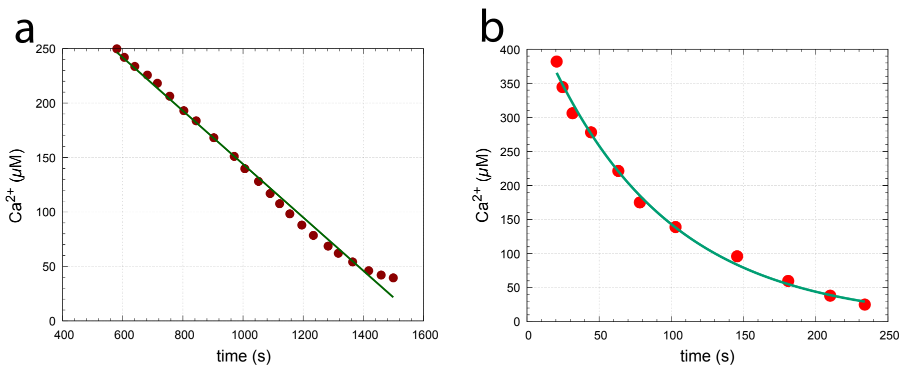

Fig S1. Decrease of calcium concentration inside the ER due to leakage. Panel a: Data extracted from Camello et al. (Fig 1B in Ref. [3]). Panel b: Extracted data from Luik et al. (Fig 1a in Ref. [4]).

The data were extracted by use of the program `GraphClick` (https://graphclick.en.softonic.com/mac) and fits were made with `gnuplot`'s `fit` function (http://www.gnuplot.info/). The original data together with the Perl scripts which call `gnuplot` and are performing the fit are located in the folders `Ca_leak_data_Camello` and `Ca_leak_data_Luik`. Perl can be downloaded from https://www.perl.org/.

To run the scripts locate the Terminal (Mac or Linux/Unix computers) or the Command Prompt (Cmd, Windows computers) to one of the folders and write:

```
perl graph_Camello_fig1b.pl
```

or

`perl graph_Ca_leak_Luik.pl`

This will create the graphs in Fig S1 and the `fit.log` file inside each folder. `fit.log` lists the determined parameter values from the iterative fit.

In case of the Camello et al. data the leak of calcium out of the ER is described by:

$$Ca(t) = Ca_0 - v \cdot (t - 600) \tag{S1}$$

where Ca_0 is the initial concentration of Ca inside the ER at time t=0, while t is time in seconds and v is the (zero-order) velocity by which calcium leaks out of the ER. The number 600 is the time in seconds by which the leak experiment was started.

The estimated parameters, which give the green line in Fig S1a are:
Ca_0=(241.838 ± 2.401)μM
v=v_{leak}=(0.244743 ± 0.004662)μM/s.

In case of the Luik et al. data the leak of calcium out of the ER is described by a first-order exponential decrease of calcium in the ER, i.e.,

$$Ca(t) = Ca_0 \cdot \mathrm{e}^{-k \cdot t} \tag{S2}$$

where Ca_0 is the initial concentration of Ca inside the ER at time t=0s, while t is the time in seconds. Parameter k is the rate constant. The estimated parameters, which give the green line in Fig S1b are:
Ca_0=(465.947 ± 10.31)μM
k=(0.0118101 ± 0.0004463)s^{-1}. In comparison with the data by Oldershaw et al. (Table 1 in [1] with k≈1min^{-1}) the Luik at al rate constant (≈0.71min^{-1}) is approximately 30% lower.
The maximum calculated leak rate from the Luik et al. data at time t=0s is: v_{leak}^{max}=k×Ca_0=0.01181×465.95μM/s=5.503μM/s.

Influence of Ca leak in the model

We refer to the model and the calculations in Fig 19d where all three feedback loops are present. Fig S2 shows the perturbation profile used in Fig 19d and in the following calculations.

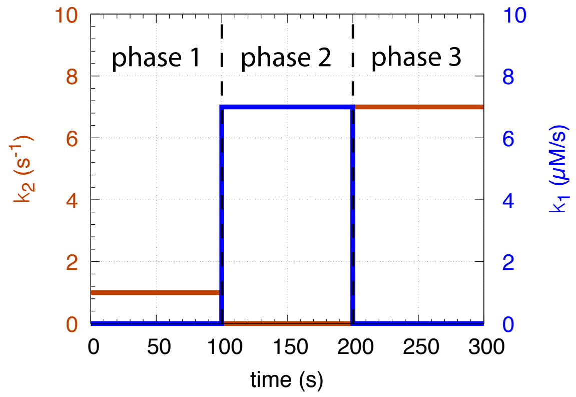

Fig S2. The perturbation profile, i.e. k_1 and k_2 values, used in Fig 19d and in the calculations of Fig S3.

Fig S3a shows a repeat of the calculation from Fig 19d, but for comparison reasons with altered ordinate axes. To allow a comparison of v_{leak} between 0.25μM/s (Camello et al. data, Fig S1a) and 5.5 μM/s (maximum v_{leak} of the Luik et al. data at t=0, Fig S1b) we have increased k_6 to 6.0μM/s, in order to avoid an uncontrolled growth in Ca_i^{2+} when $v_{leak}>k_6$.

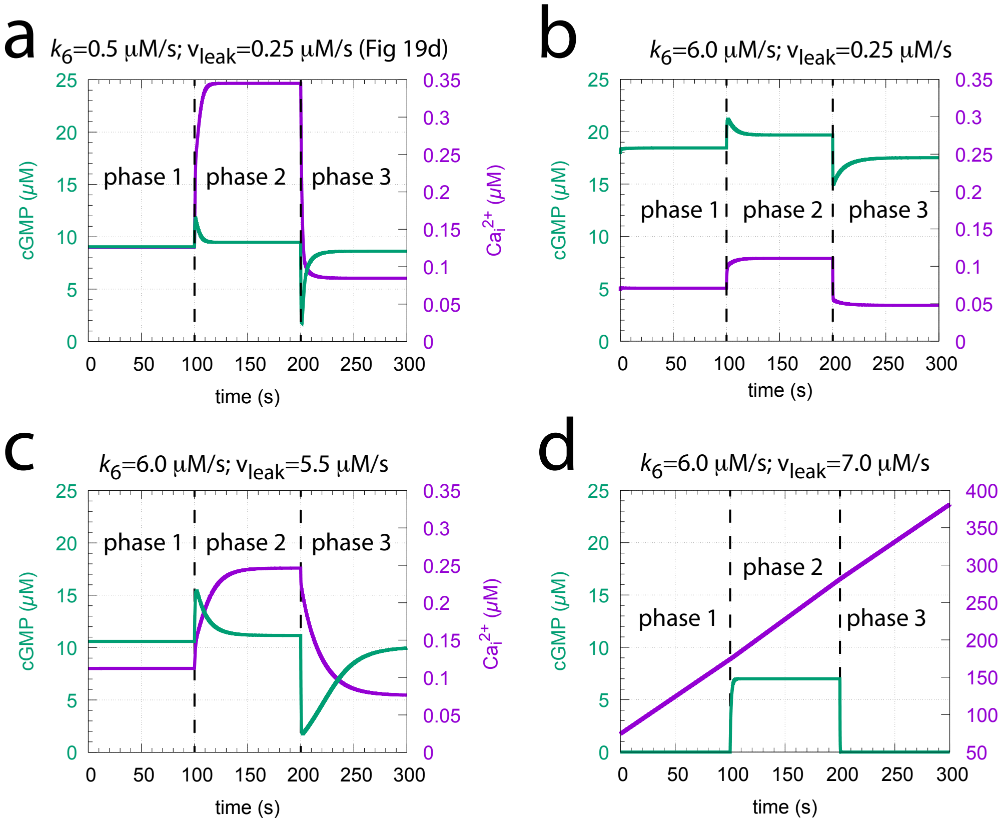

Fig S3. Influence of cGMP and Ca_i^{2+} homeostasis with respect to the perturbation profile in Fig S2 and as a function of v_{leak}. (a) Identical to Fig 19d, but with altered ordinate axes (for comparison with the other panels). v_{leak}=0.25μM/s as determined by the Camello et al. data above. Initial concentrations: cGMP=9.042μM, Ca_i^{2+}=125.7 nM, K^+=1.989μM. (b) As (a), but k_6 is increased to 6.0μM/s. Initial concentrations: cGMP=17.862μM, Ca_i^{2+}=72.96 nM, K^+=41.119μM. (c) As (b), but v_{leak}=5.5μM/s, i.e. the maximum value obtained from the Luik et al. data above. Initial concentrations: cGMP=10.592μM, Ca_i^{2+}=112.10 nM, K^+=26.762μM. (d) As (c), but v_{leak}=7.0μM/s ($>k_6$). Initial concentrations as in panel c. Other rate constants (panels a-d): k_3=50μM/s, k_4 (background) = 0s^{-1}, k_5=100.0μM, k_7=2.0$\mu M^{-1}s^{-1}$, k_8=5.75×$10^{-2}\mu$M, r=1.65, k_9=1.0s^{-1}, k_{10}=6.36×$10^{-2}\mu$M, m=2.5, k_{11}=32.8 μM, n=4.1, k_{12}=0.623μM, p=0.894.

Fig S1c shows the behavior of the system when v_{leak} is 5.5 μM/s, i.e. the maximum value as calculated above by the Luik et al. data. As expected the Ca_i^{2+} level has increased, and as a result of this, the Ca_i^{2+} inhibition term in Eq 21 leads to a decrease of the cGMP concentration in comparison with Fig S1b. Finally, when v_{leak}=7.0 μM/s and larger than k_6, Ca_i^{2+} increases uncontrolled as shown in Fig S1d. The negative feedback loops through cGMP are broken as the Ca_i^{2+}-activated removal of cGMP in feedback loop 2 becomes saturated and the Ca_i^{2+}-inhibited synthesis rate of cGMP in

feedback loop 1 goes to zero.

References

1. Oldershaw KA, Nunn D, Taylor CW. Quantal Ca^{2+} mobilization stimulated by inositol 1, 4, 5-trisphosphate in permeabilized hepatocytes. Biochemical Journal. 1991;278(3):705–708.

2. Missiaen L, Smedt HD, Parys JB, Raeymaekers L, Droogmans G, Bosch LVD, et al. Kinetics of the non-specific calcium leak from non-mitochondrial calcium stores in permeabilized A7r5 cells. Biochemical Journal. 1996;317(3):849–853.

3. Camello C, Lomax R, Petersen OH, Tepikin A. Calcium leak from intracellular stores—the enigma of calcium signalling. Cell Calcium. 2002;32(5-6):355–361.

4. Luik RM, Wang B, Prakriya M, Wu MM, Lewis RS. Oligomerization of STIM1 couples ER calcium depletion to CRAC channel activation. Nature. 2008;454(7203):538–542.
