# Supplementary figures and images for: Homeostasis at different backgrounds: The roles of overlayed feedback structures in vertebrate photoadaptation
Source: PLoS One. 2023 Apr 28;18(4):e0281490. doi: 10.1371/journal.pone.0281490 (PMC10146485; doi:10.1371/journal.pone.0281490)

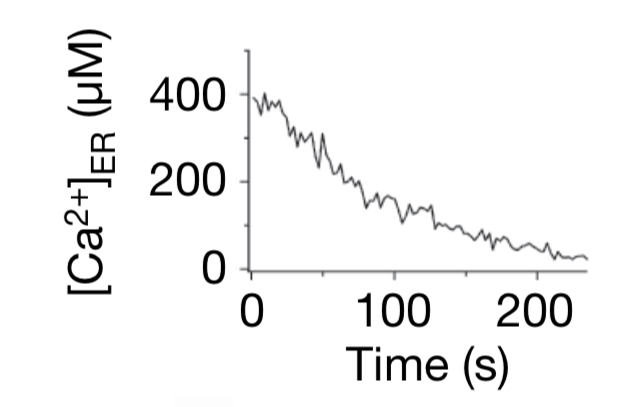

Supplement: S4 Text — A comparison how experimentally observed zero-order and first-order Ca leak kinetics affect photoadaptation in the model and when homeostatic breakdown occurs. (ZIP) [file pone.0281490.s005.zip › Ca_leak_data_Luik/original data.png]

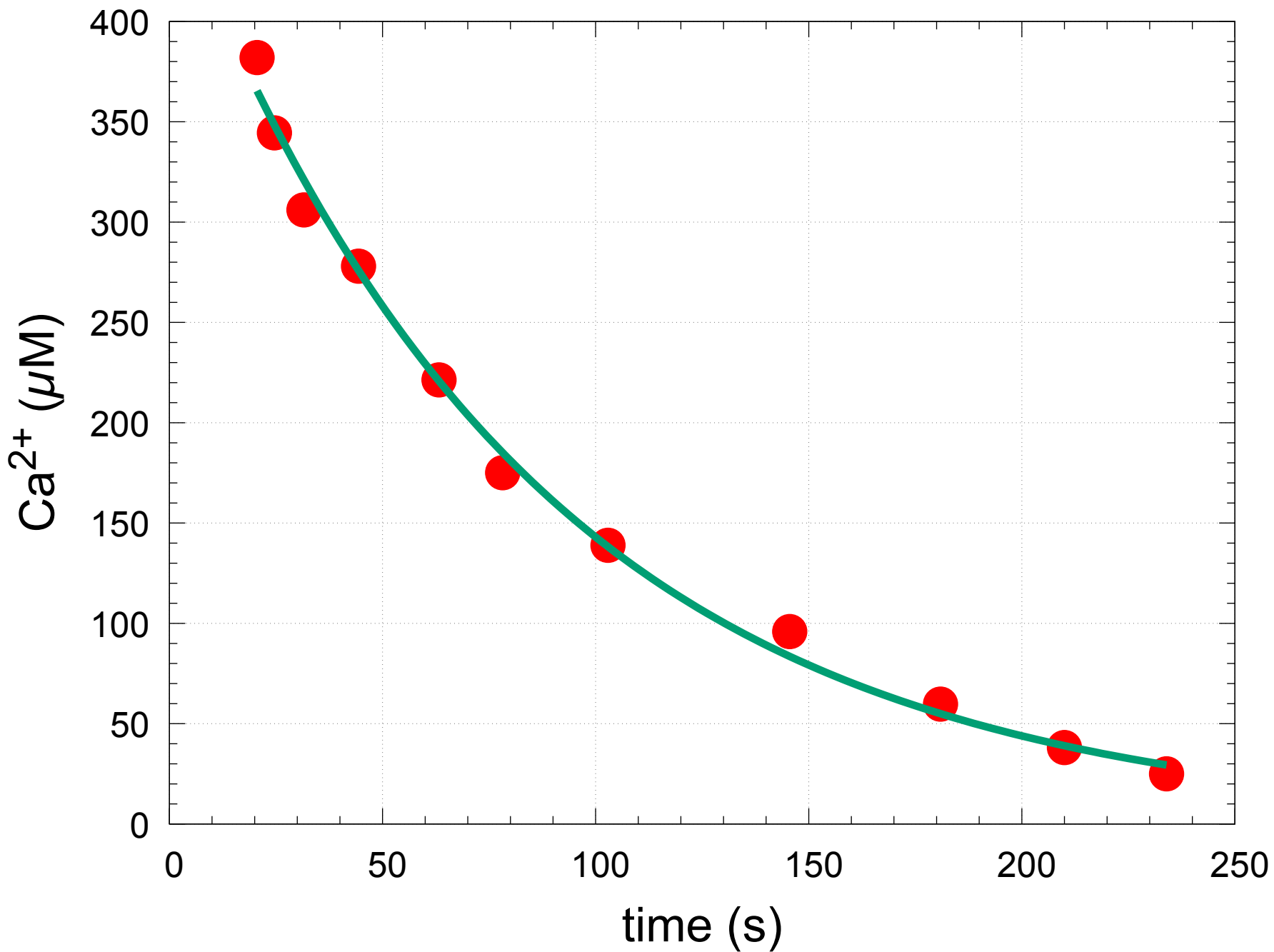

Supplement: S4 Text — A comparison how experimentally observed zero-order and first-order Ca leak kinetics affect photoadaptation in the model and when homeostatic breakdown occurs. (ZIP) [file pone.0281490.s005.zip › Ca_leak_data_Luik/graph_Ca_leak_Luik.pdf]

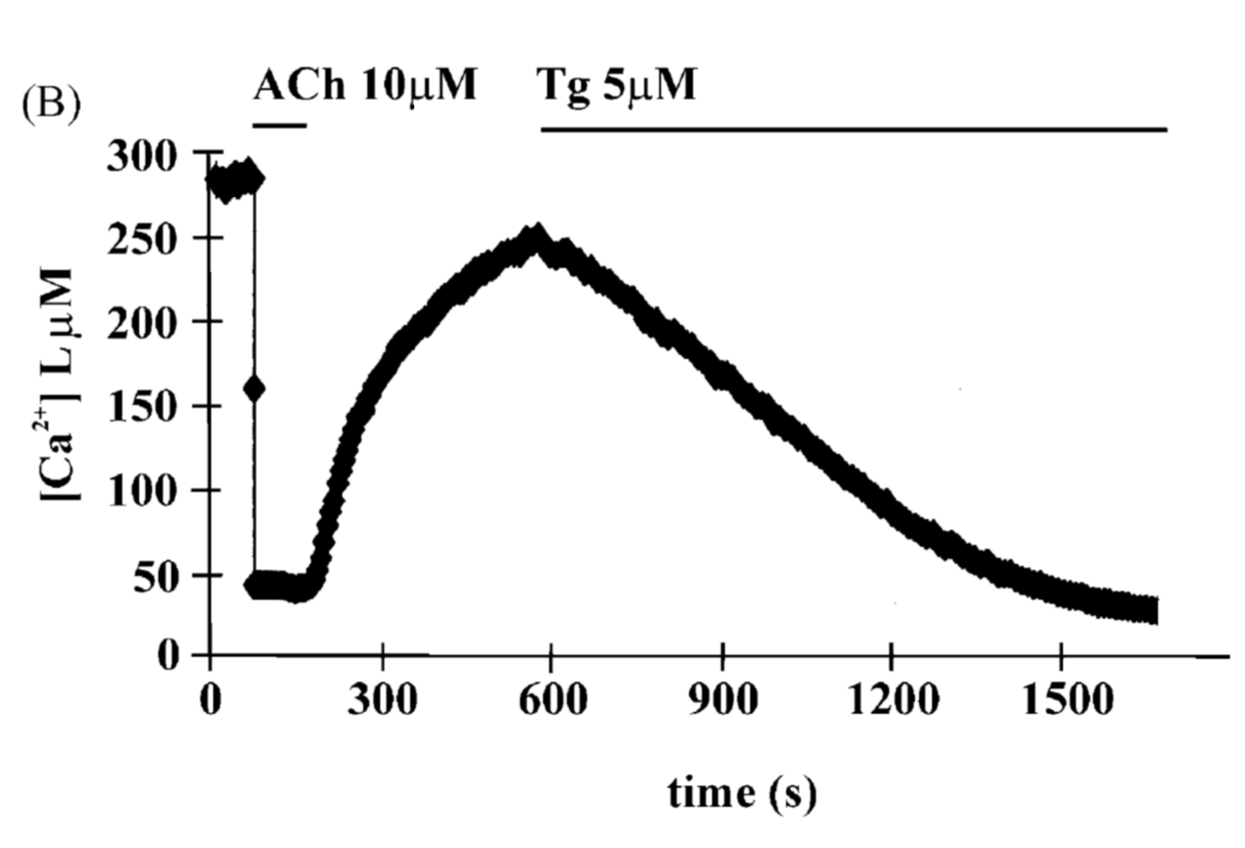

Supplement: S4 Text — A comparison how experimentally observed zero-order and first-order Ca leak kinetics affect photoadaptation in the model and when homeostatic breakdown occurs. (ZIP) [file pone.0281490.s005.zip › Ca_leak_data_Camello/original data.png]

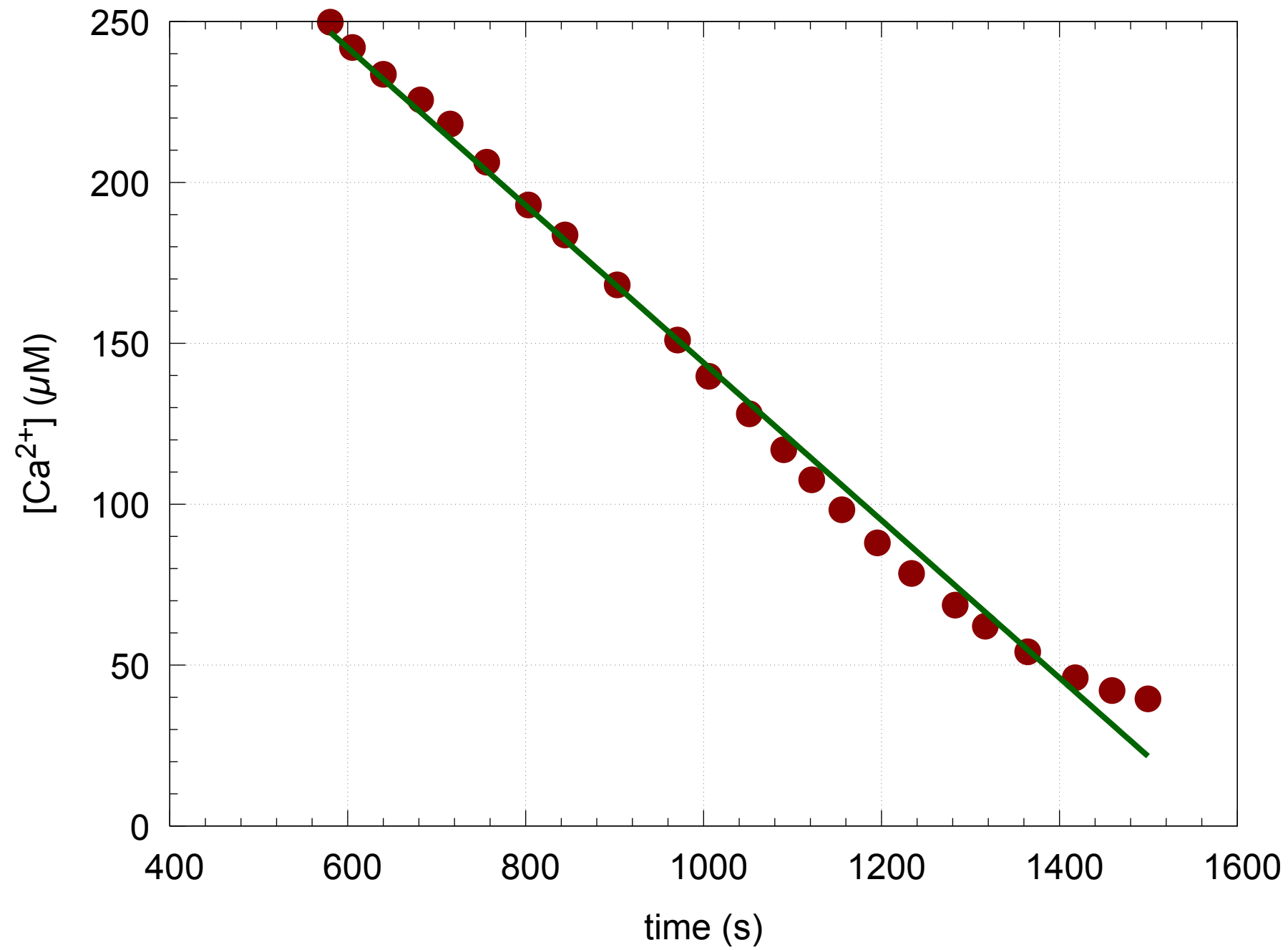

Supplement: S4 Text — A comparison how experimentally observed zero-order and first-order Ca leak kinetics affect photoadaptation in the model and when homeostatic breakdown occurs. (ZIP) [file pone.0281490.s005.zip › Ca_leak_data_Camello/graph_Ca-leak_data_Camillo_fig1b.pdf]

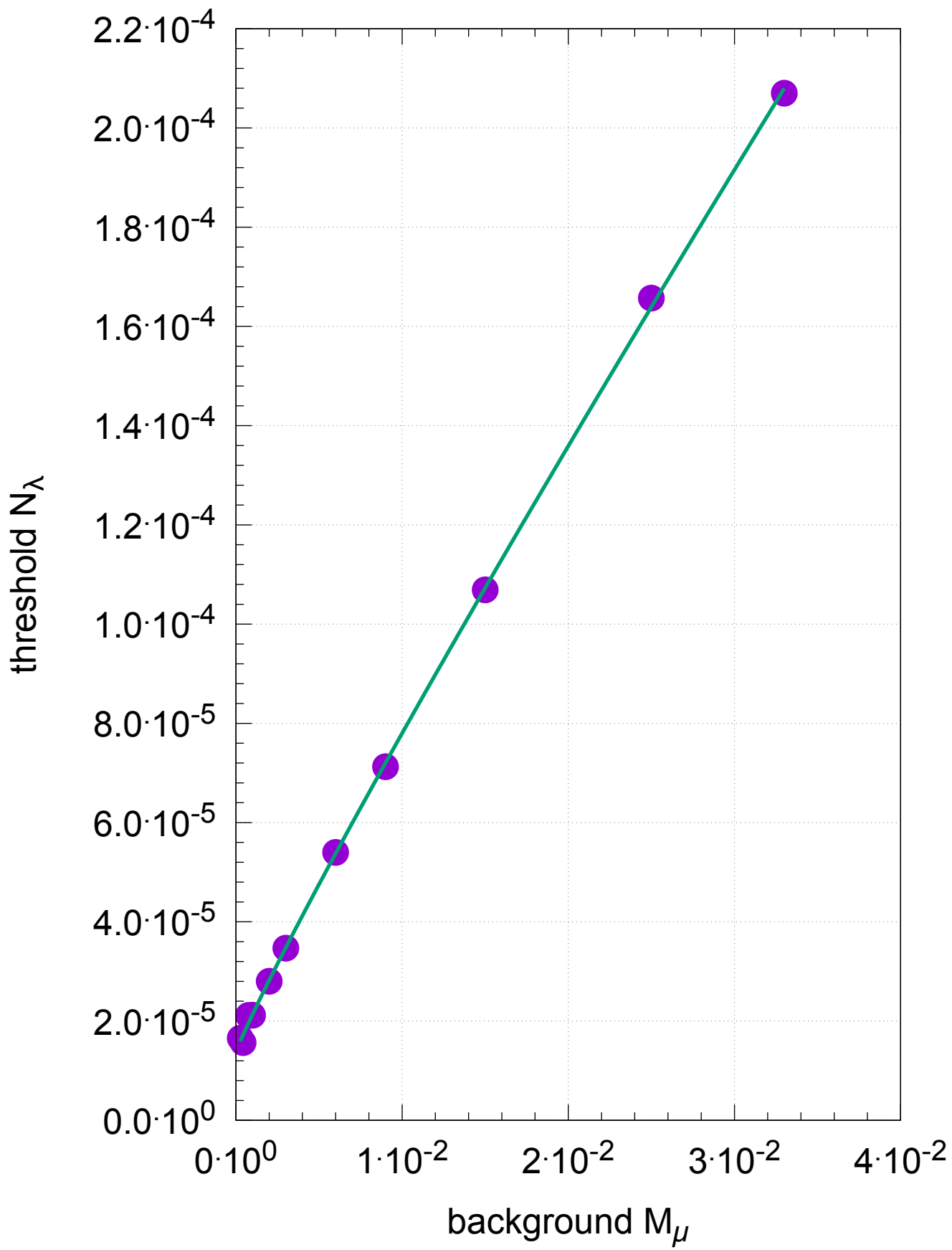

Supplement: S6 Text — Replots of experimental data show, as indicated by model calculations, that Stephens’ law is followed at low backgrounds, while at higher backgrounds the response tends towards Weber’s law. (ZIP) [file pone.0281490.s007.zip › S6 Text/green_blue/graph_blue_lin.pdf]

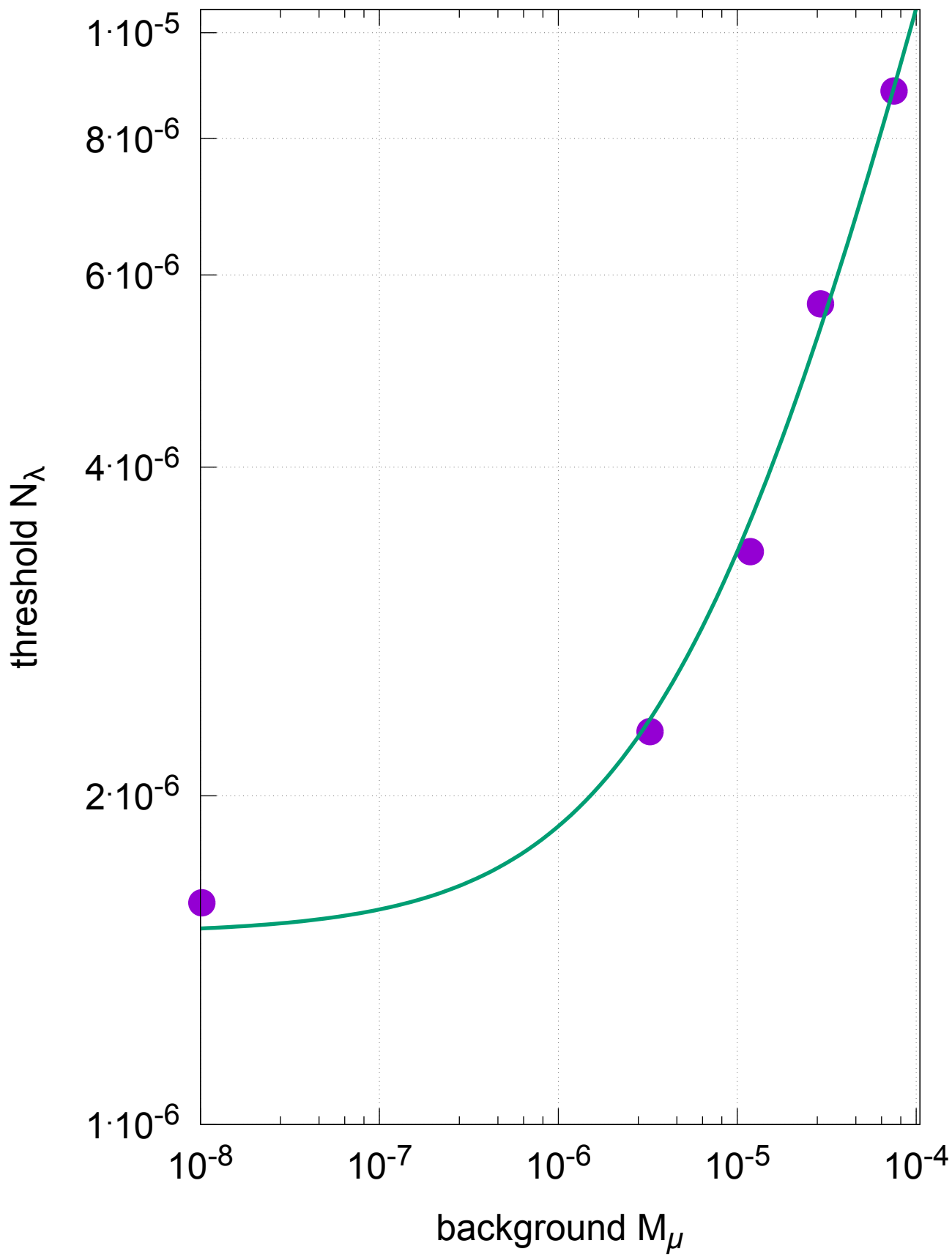

Supplement: S6 Text — Replots of experimental data show, as indicated by model calculations, that Stephens’ law is followed at low backgrounds, while at higher backgrounds the response tends towards Weber’s law. (ZIP) [file pone.0281490.s007.zip › S6 Text/green_blue/graph_green_log.pdf]

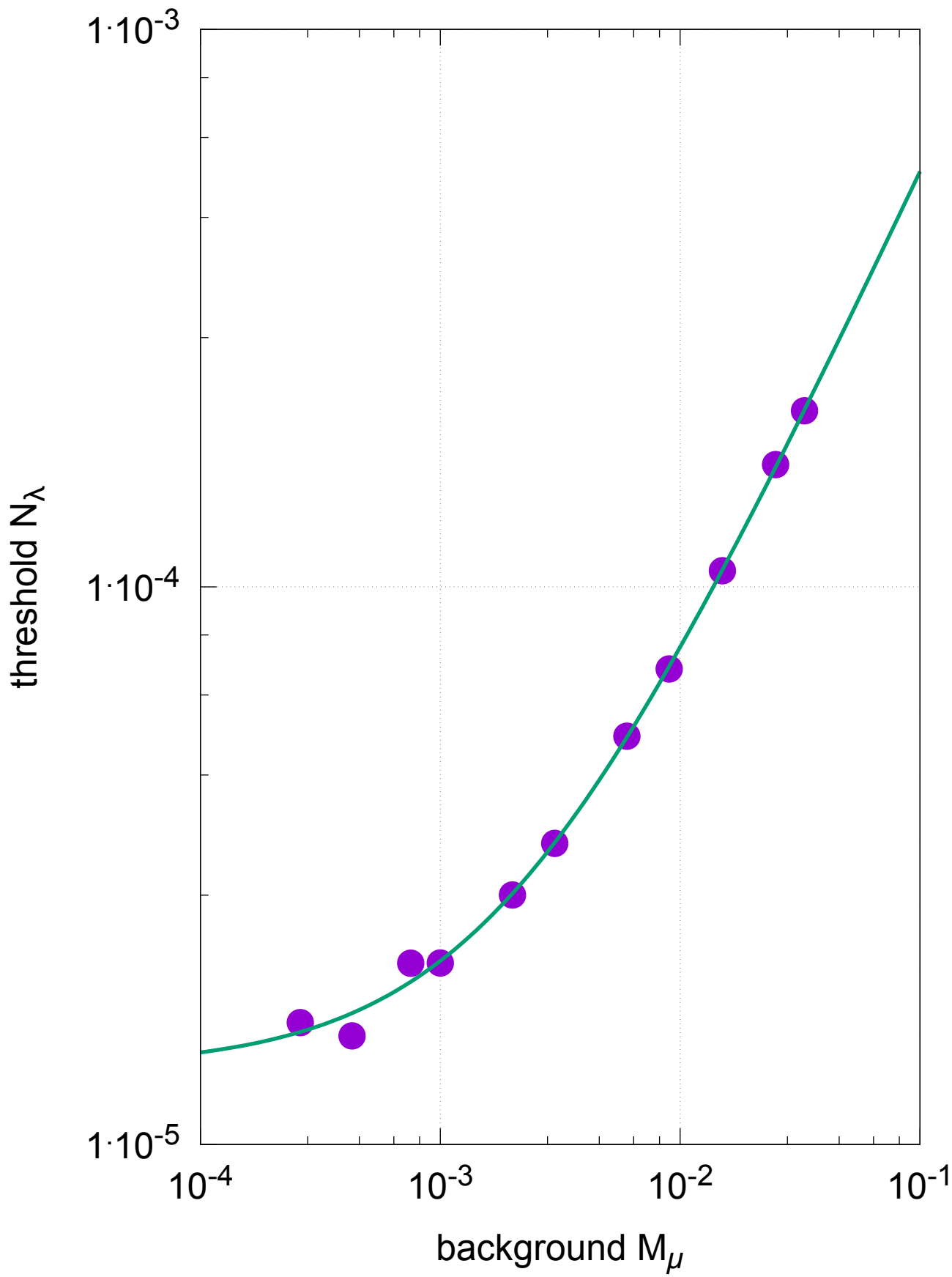

Supplement: S6 Text — Replots of experimental data show, as indicated by model calculations, that Stephens’ law is followed at low backgrounds, while at higher backgrounds the response tends towards Weber’s law. (ZIP) [file pone.0281490.s007.zip › S6 Text/green_blue/graph_blue_log.pdf]

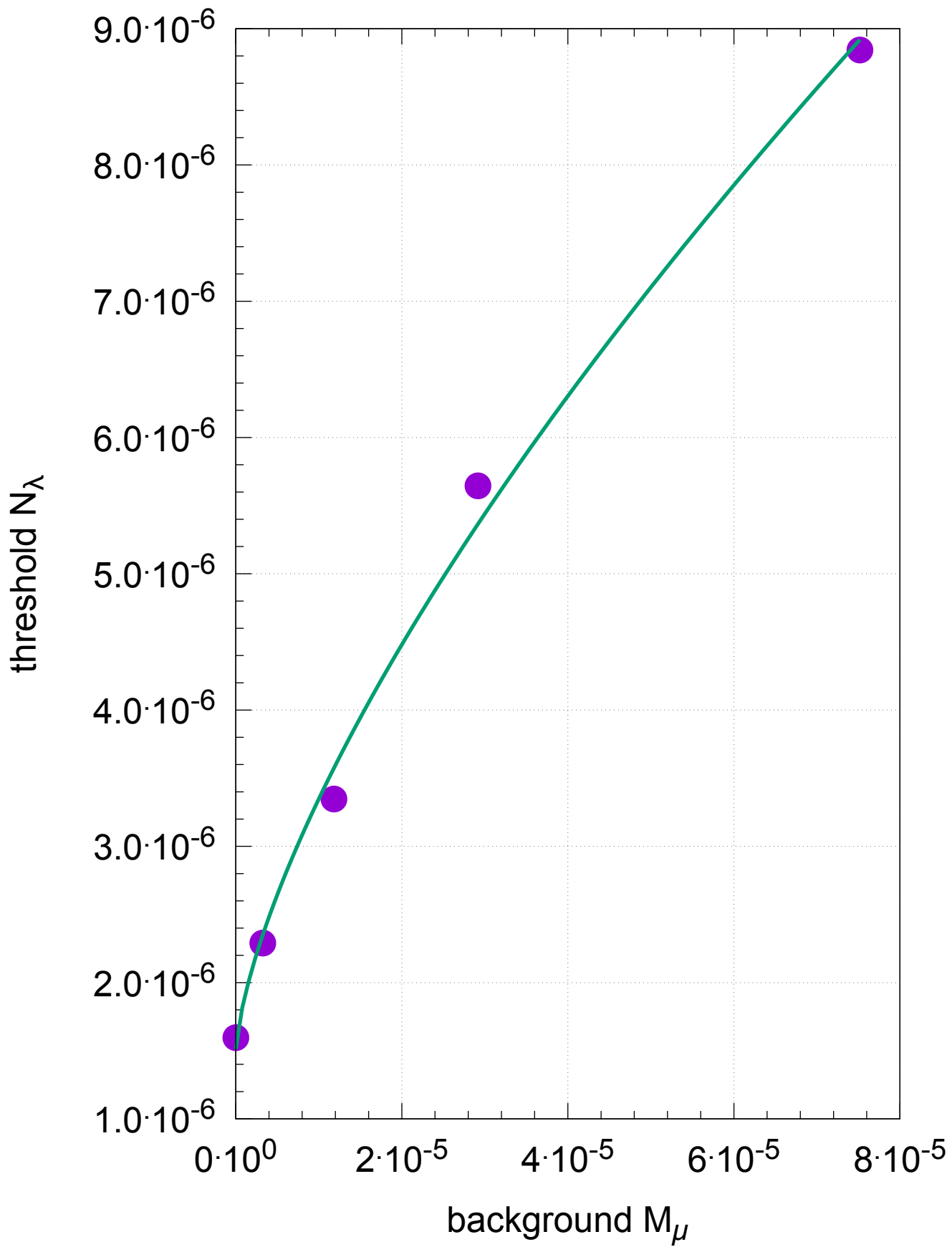

Supplement: S6 Text — Replots of experimental data show, as indicated by model calculations, that Stephens’ law is followed at low backgrounds, while at higher backgrounds the response tends towards Weber’s law. (ZIP) [file pone.0281490.s007.zip › S6 Text/green_blue/graph_green_lin.pdf]

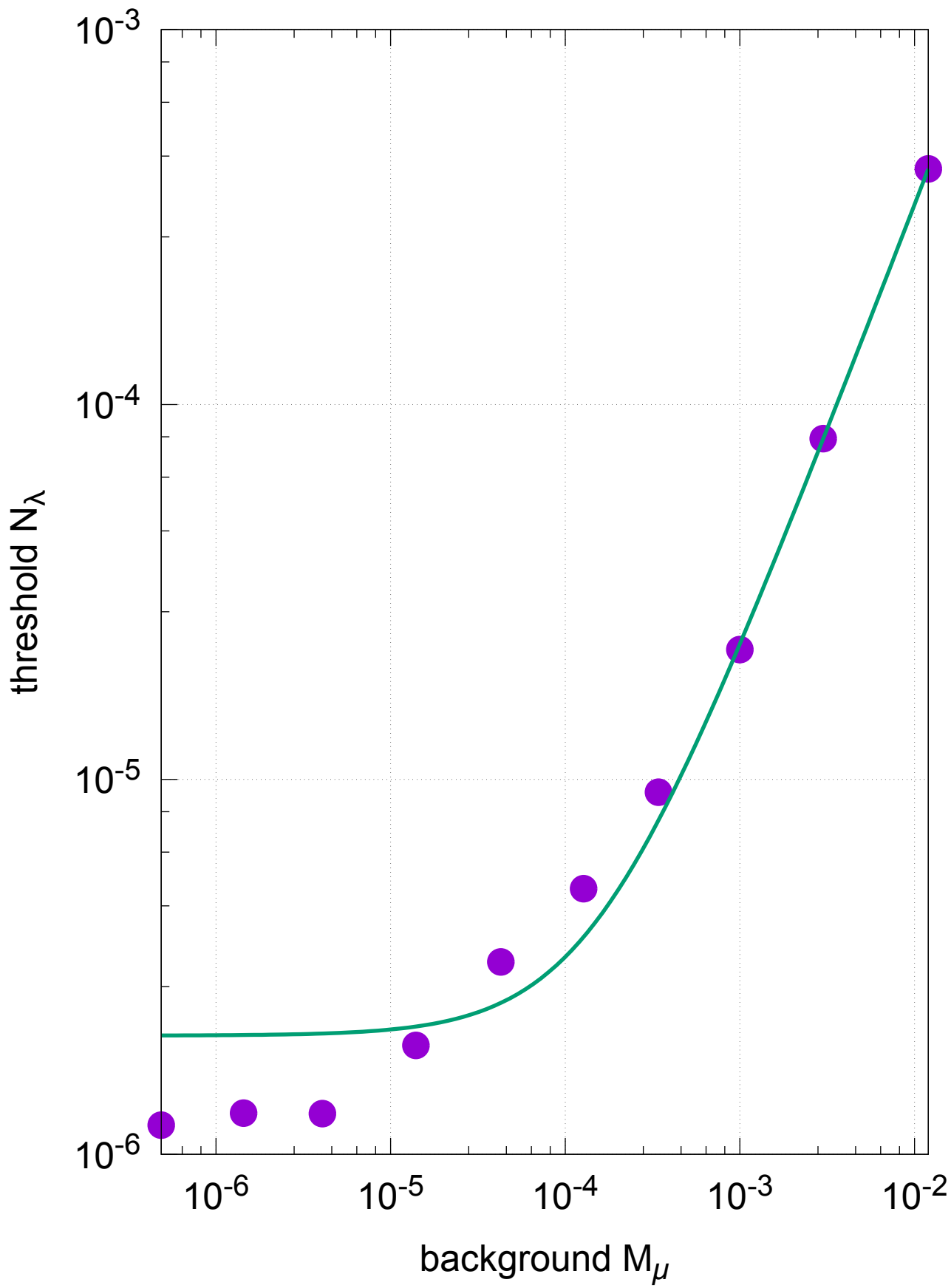

Supplement: S6 Text — Replots of experimental data show, as indicated by model calculations, that Stephens’ law is followed at low backgrounds, while at higher backgrounds the response tends towards Weber’s law. (ZIP) [file pone.0281490.s007.zip › S6 Text/rod_cones/graph_cones_log.pdf]

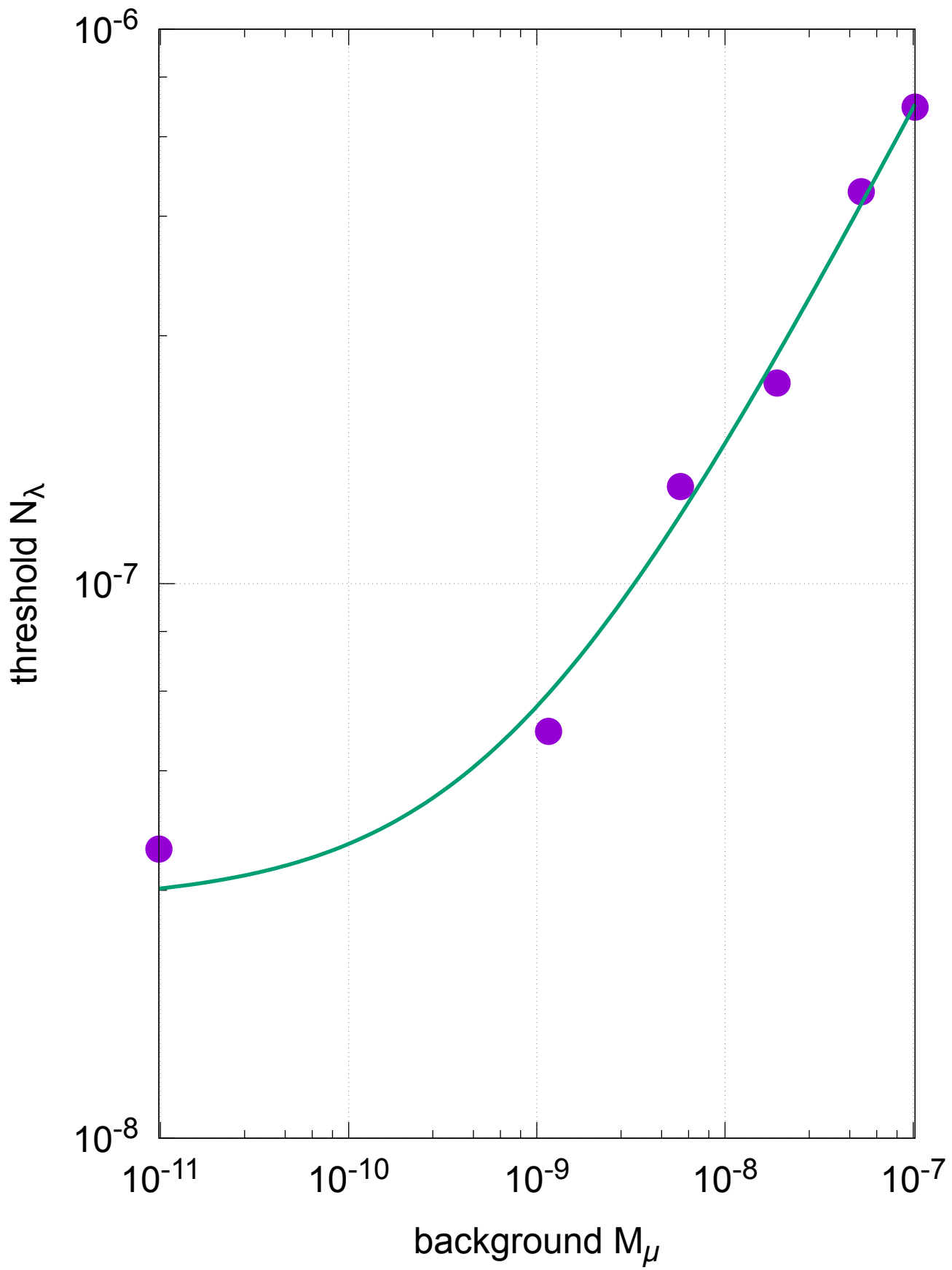

Supplement: S6 Text — Replots of experimental data show, as indicated by model calculations, that Stephens’ law is followed at low backgrounds, while at higher backgrounds the response tends towards Weber’s law. (ZIP) [file pone.0281490.s007.zip › S6 Text/rod_cones/graph_rods_log.pdf]

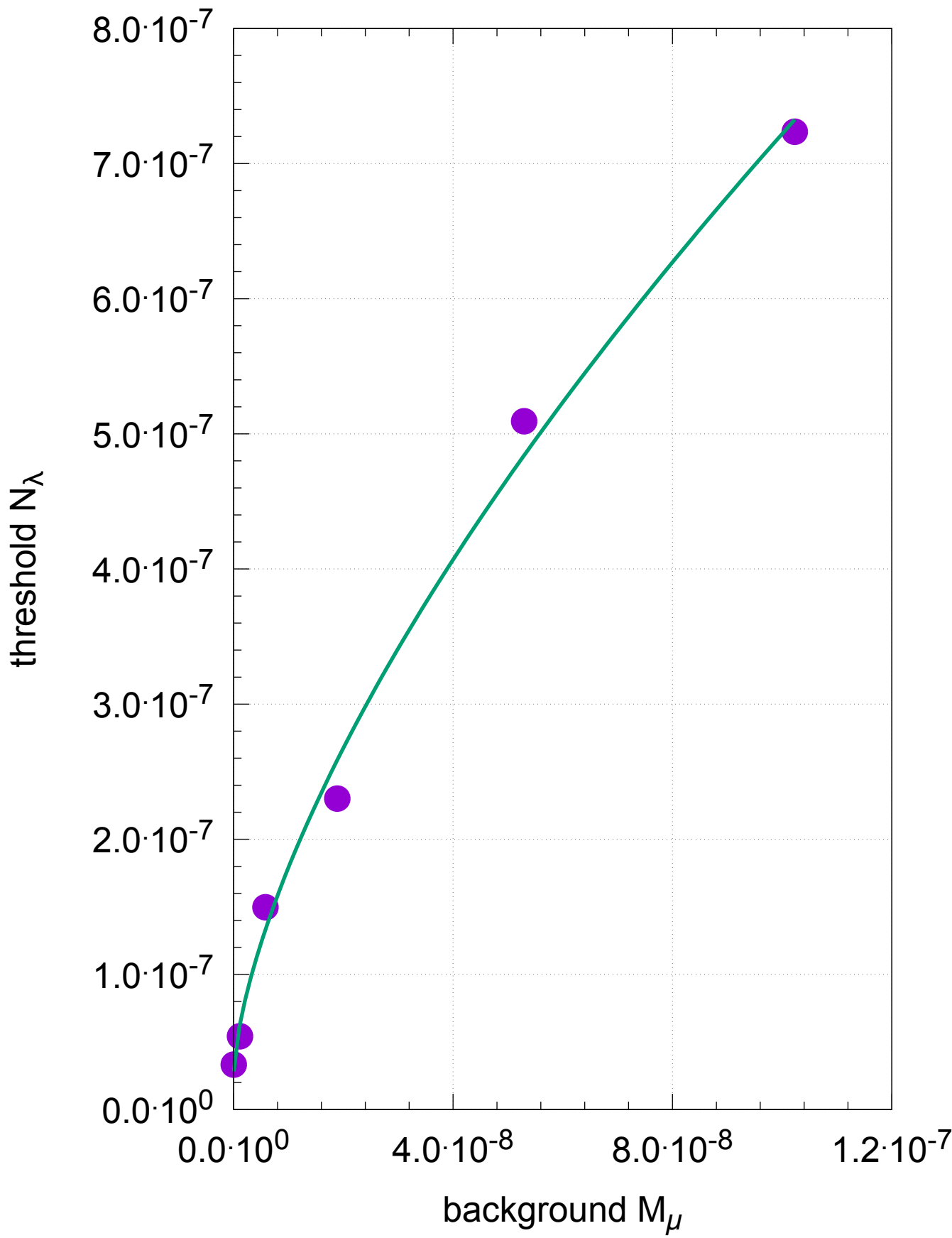

Supplement: S6 Text — Replots of experimental data show, as indicated by model calculations, that Stephens’ law is followed at low backgrounds, while at higher backgrounds the response tends towards Weber’s law. (ZIP) [file pone.0281490.s007.zip › S6 Text/rod_cones/graph_rods_lin.pdf]

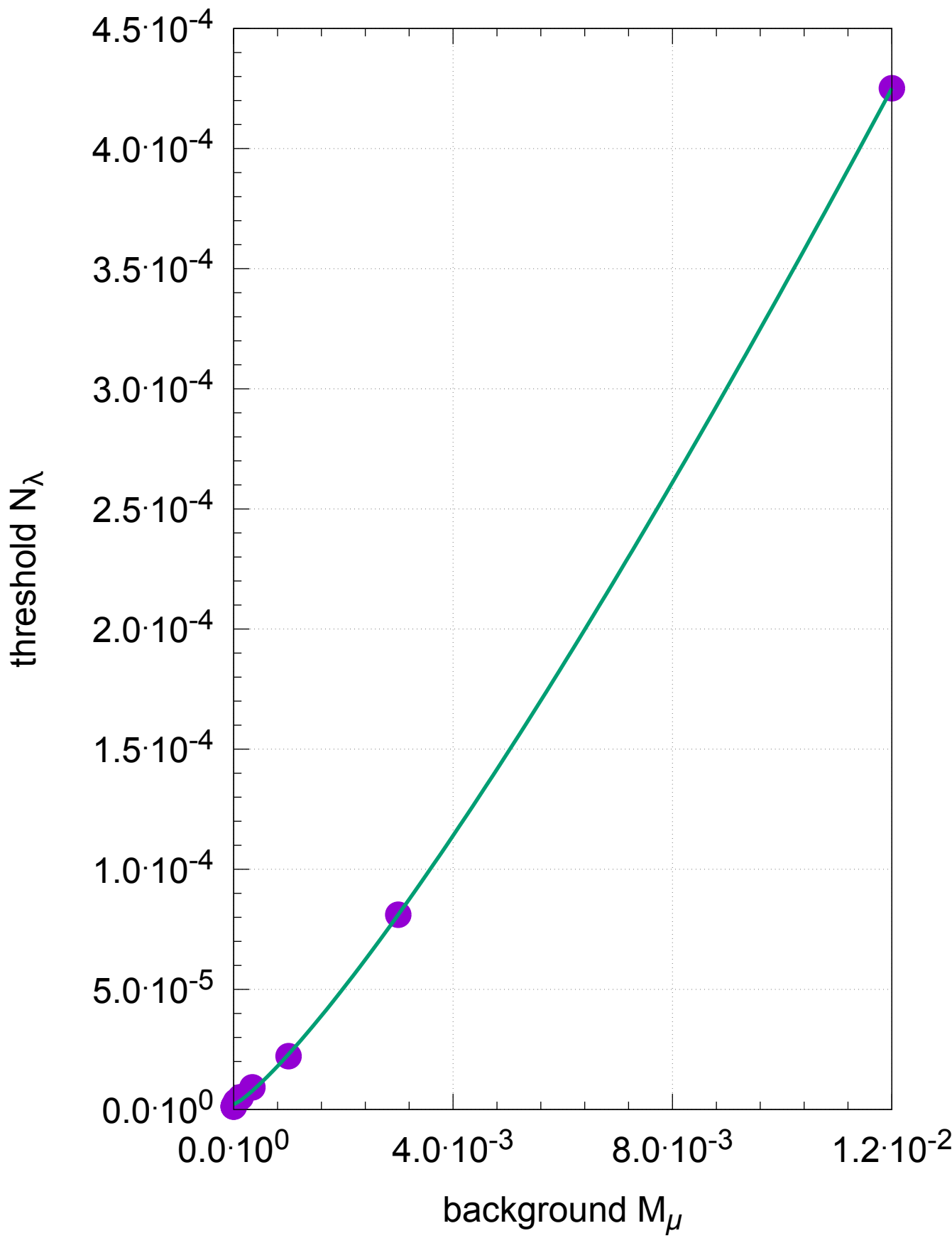

Supplement: S6 Text — Replots of experimental data show, as indicated by model calculations, that Stephens’ law is followed at low backgrounds, while at higher backgrounds the response tends towards Weber’s law. (ZIP) [file pone.0281490.s007.zip › S6 Text/rod_cones/graph_cones_lin.pdf]
