# Supplementary material for: Homeostasis at different backgrounds: The roles of overlayed feedback structures in vertebrate photoadaptation
Source: PLoS One. 2023 Apr 28;18(4):e0281490. doi: 10.1371/journal.pone.0281490 (PMC10146485; doi:10.1371/journal.pone.0281490)
Supplement: S5 Text — By using a k1-k2 perturbation profile influences of k5, k6, and k7 on the model’s resetting kinetics are shown. (ZIP) [file pone.0281490.s006.zip › S5 Text.pdf]

Homeostasis at different backgrounds: The roles of overlayed feedback structures in vertebrate photoadaptation

Jonas V. Grini, Melissa Nygård, Peter Ruoff*

Department of Chemistry, Bioscience, and Environmental Engineering, University of Stavanger, Stavanger, Norway

Supporting Information S5 Text

Influence of k_5, k_6, and k_7 on the model's photoadaptation

In the three figures below all panels (a) show the applied k_1/k_2 perturbation profile while all panels (b) show the response of a reference state identical to that shown in Fig 19d. Panels c and d show the responses when parameters are changed.

Influence of k_5

In comparison with the reference, an increase of k_5 lowers the levels of cGMP, but

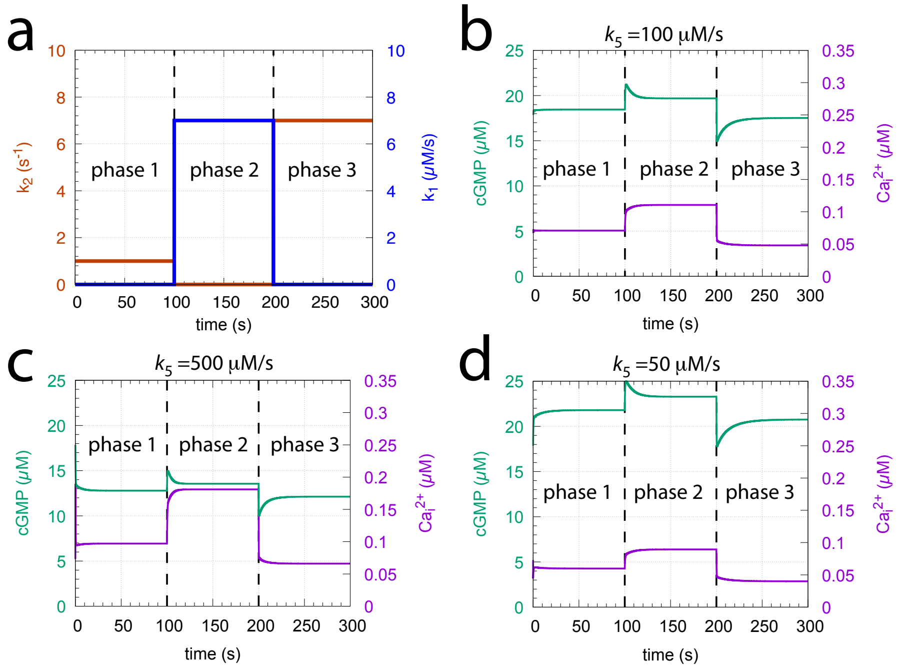

Fig S1. Influence of k_5 on the model's photoadaptation response. (a) Perturbation profile. The profile is identical to that applied in Fig 19d and shown in Fig S2 in Supporting Information S4 Text. (b) Reference state. Rate constants and initial concentrations are the same as in Fig 19d and in Fig S3b (see Supporting Information S4 Text). In the reference state we have that k_5=100 μM/s. (c) As reference, but k_5=500 μM/s. (d) As reference, but k_5=50 μM/s.

increases Ca_i^{2+} concentrations. When k_5 is decreased cGMP levels increase and Ca_i^{2+} concentrations decrease slightly.

Influence of k_6

Changes in k_6 are "opposite" to changes in k_5, i.e., an increase in k_6 from 6.0 μM/s (Fig S2b) to 12.0 μM/s (Fig S2c) increases the level of cGMP, but decreases Ca_i^{2+} concentrations slightly, while a decrease from 6.0 μM/s to 0.5 μM/s shows a decrease in cGMP concentrations and a relative large increase in Ca_i^{2+} when k_1 is high (Fig S2d). It should also be noted that when $v_{leak}>k_6$ the model's adaptation breaks down and Ca_i^{2+} growths uncontrolled (see Fig S3d in Supporting Information S4 Text).

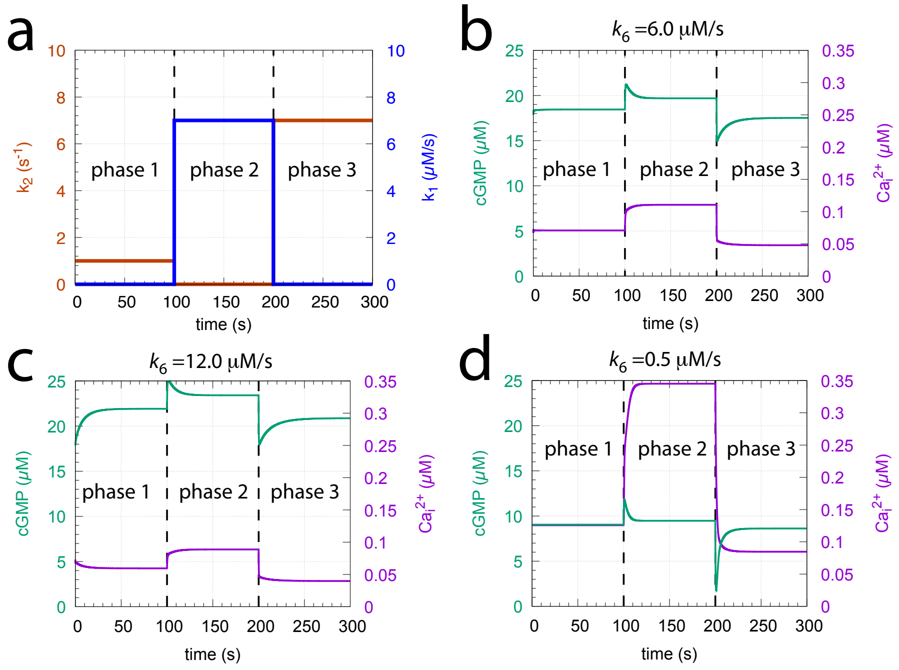

Fig S2. Influence of k_6 on the model's photoadaptation response. (a) Perturbation profile. The profile is identical to that applied in Fig 19d and shown in Fig S2 in Supporting Information S4 Text. (b) Reference state. Rate constants and initial concentrations are the same as in Fig 19d and in Fig S3b (see Supporting Information S4 Text). In the reference state we have that k_6=6.0 μM/s. (c) As reference, but k_6=12 μM/s. (d) As reference, but k_6=0.5 μM/s.

Influence of k_7

Changes in k_7 have no influence on the steady state levels of cGMP and Ca_i^{2+}, but on their resetting kinetics, i.e. how fast the steady state levels are reached. When k_7 is higher than the reference, then the steady states are approached faster (Fig S3c). On the other hand, when k_7 is lower than the reference, the approach to the steady states is slower (Fig S3d).

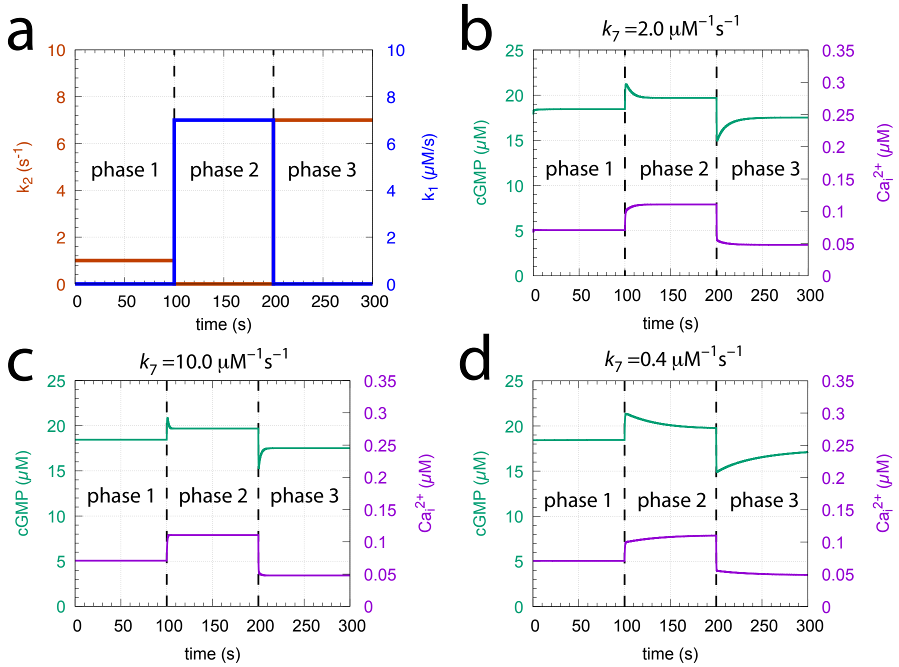

Fig S3. Influence of k_6 on the model's photoadaptation response. (a) Perturbation profile. The profile is identical to that applied in Fig 19d and shown in Fig S2 in Supporting Information S4 Text. (b) Reference state. Rate constants and initial concentrations are the same as in Fig 19d and in Fig S3b in Supporting Information S4 Text. In the reference state we have that k_7=2.0μM^{-1}s^{-1}. (c) As reference, but k_7=10.0 μM^{-1}s^{-1}. (d) As reference, but k_7=0.4 μM^{-1}s^{-1}.
