# Supplementary material for: Homeostasis at different backgrounds: The roles of overlayed feedback structures in vertebrate photoadaptation
Source: PLoS One. 2023 Apr 28;18(4):e0281490. doi: 10.1371/journal.pone.0281490 (PMC10146485; doi:10.1371/journal.pone.0281490)
Supplement: S6 Text — Replots of experimental data show, as indicated by model calculations, that Stephens’ law is followed at low backgrounds, while at higher backgrounds the response tends towards Weber’s law. (ZIP) [file pone.0281490.s007.zip › S6 Text/S6 Text.pdf]

Homeostasis at different backgrounds: The roles of overlayed feedback structures in vertebrate photoadaptation

Jonas V. Grini, Melissa Nygård, Peter Ruoff*

Department of Chemistry, Bioscience, and Environmental Engineering, University of Stavanger, Stavanger, Norway

Supporting Information S6 Text

Experimental light adaptation data

To see whether the model's response on pulse perturbations (Fig 15) describe qualitatively experimental results, we have taken experimental *threshold versus background radiance* data by Wyszecki and Stiles (Fig 2(7.4.1) and Fig 3(7.4.1) in Ref. [1]) and reanalyzed these data in terms of a Stephens' based power-law of the form

$$N_\lambda = \alpha \cdot M_\mu^n + N_{\lambda,0} \tag{S1}$$

N_λ is the threshold light intensity (radiance) at wavelength λ, and M_μ is the background light intensity at wavelength μ. α, n, and $N_{\lambda,0}$ are adjustable parameters.

Data extraction and fitting procedure

The experimental data were extracted from Fig 2(7.4.1) and Fig 3(7.4.1) in Ref. [1] by using `GraphClick for Mac` (https://graphclick.en.softonic.com/mac). Eq S1 was fitted to the experimental data by `gnuplot`'s fit function, which is based on the Levenberg-Marquardt algorithm (see gnuplot documentation at http://www.gnuplot.info/). To redo these fits by the reader, we have provided `Perl` scripts (https://www.perl.org/) together with the extracted experimental data. Please, see the description below on how to run the scripts.

Rod and cone branches

Figs S1 and S2 show the replotted data from Fig 2(7.4.1) of Ref. [1]) together with the fit by Eq S1 (green lines). Fig S1a shows that rods at low background intensities are better described by Stephens' power-law model with n around 0.66 than by Weber's law, which would require a n of 1.

On the other hand, at higher background intensities, the cone branch comes closer to a linear description by Weber's law with a n of about 1.21.

This is qualitatively in accordance with the model findings (Fig 15), which at low intensities show a n of 0.8, while at higher intensities n is close to one (1.01).

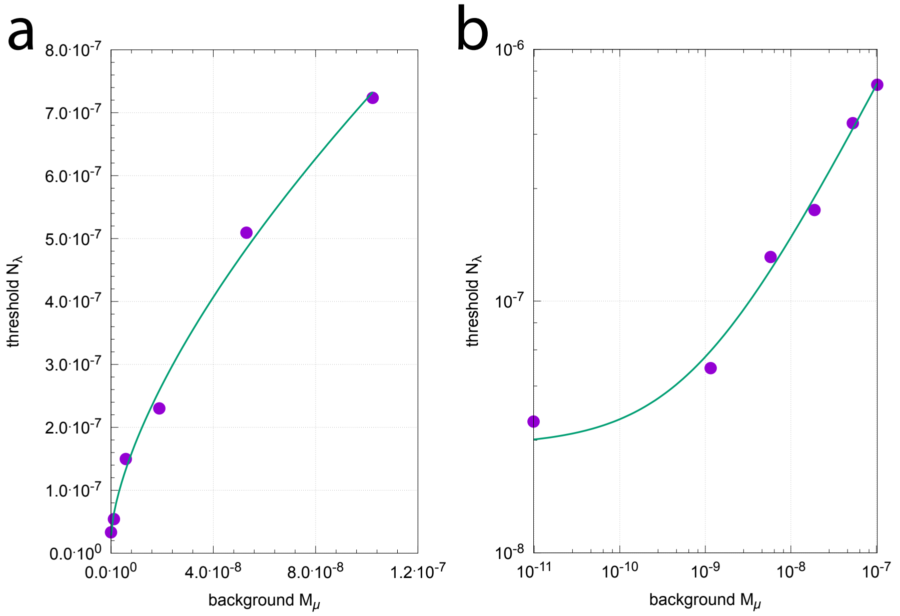

Fig S1. 5° parafoveal light adaptation of the retina's rod branch for λ=580 nm and μ=500 nm (replotted from Fig 2(7.4.1) of Ref. [1]). Panels a and b show the same fit, but with linear or log-log axes, respectively. The parameter values with asymptotic standard errors are: α=0.0284193 $\pm$ 0.03201, n=0.658764 $\pm$ 0.07072, and $N_{\lambda,0} = 2.66026{\times}10^{-08} \pm 2.132{\times}10^{-08}$.

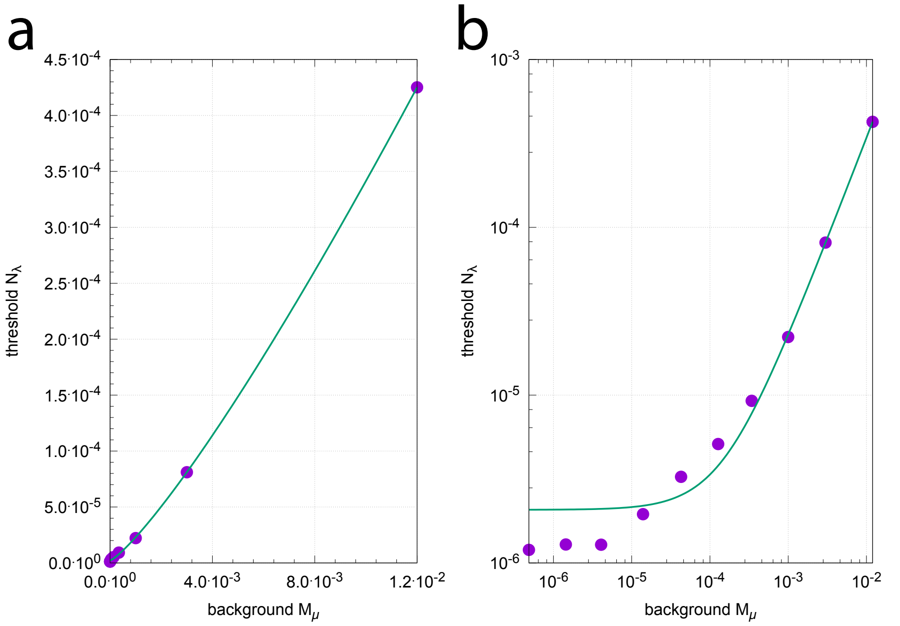

Fig S2. 5° parafoveal light adaptation of the retina's cone branch for λ=580 nm and μ=500 nm (replotted from Fig 2(7.4.1) of Ref. [1]). Panels a and b show the same fit, but with linear or log-log axes, respectively. The parameter values with asymptotic standard errors are: α=0.0893037 $\pm$ 0.003747, n=1.21017 $\pm$ 0.0095, and $N_{\lambda,0} = 2.07317{\times}10^{-06} \pm 3.924{\times}10^{-07}$.

Green and blue sensitive cone branches

Figs S3 and S4 show the replots of Fig 3(7.4.1) [1] for the foveal green-sensitive and blue-sensitive cone mechanisms, respectively.

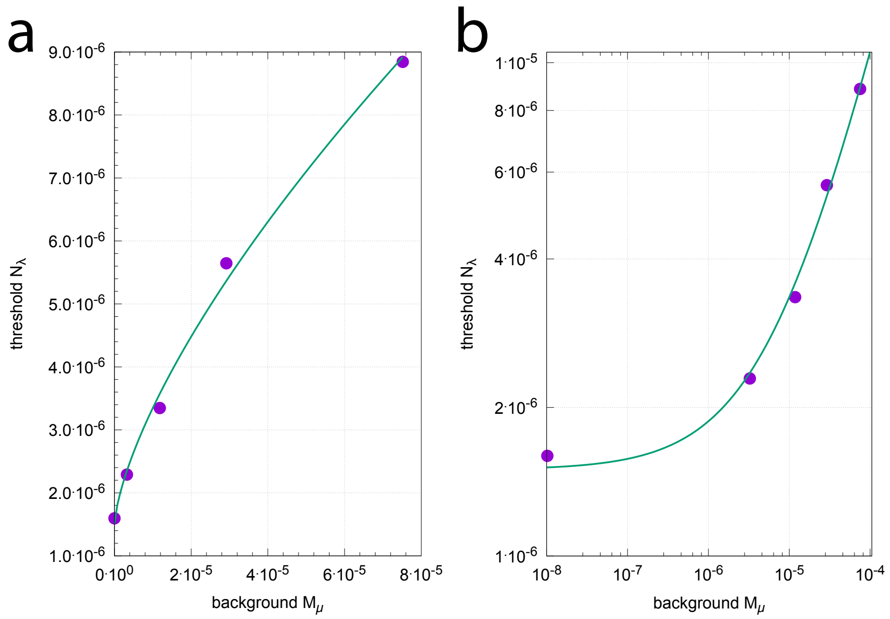

Fig S3. Foveal green cone sensitive threshold versus background data at λ=475 nm and μ=550 nm. Panels a and b show the same fit to Eq S1 (green lines), but with linear or log-log axes, respectively. The parameter values with asymptotic standard errors are: α=0.00512025 $\pm$ 0.003573, n=0.688351 $\pm$ 0.07469, and $N_{\lambda,0} = 1.49602\times10^{-06} \pm 2.562\times10^{-07}$.

Also in this case low background intensities show a green cone adaptation behavior towards Stephens' power-law with n around 0.69, while at higher intensities the blue cone adaptation is closer to Weber's linear law with n at about 0.93.

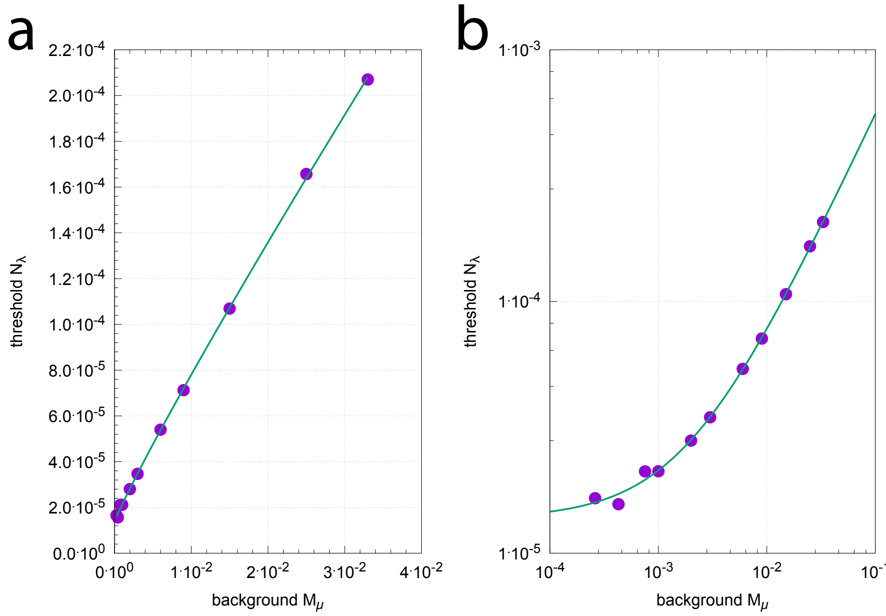

Fig S4. Foveal blue cone sensitive threshold versus background data at λ=475 nm and μ=550 nm. Panels a and b show the same fit to Eq S1 (green lines), but with linear or log-log axes, respectively. The parameter values with asymptotic standard errors are: α=0.00457834 $\pm$ 0.0002134, n=0.926321 $\pm$ 0.01364, and $N_{\lambda,0} = 1.37232{\times}10^{-05} \pm 6.961{\times}10^{-07}$.

How to run the scripts

The supporting information contains the two folders `rod_cones` and `green_blue`. In these folders the extracted numerical data are found in the files with extension `.txt`.

The files with extension `.pl` contain the Perl scripts, while the files with extension `.log` contain information about the fit, i.e. the optimized parameter values together with their asymptotic standard errors.

The `pdf` files show the generated plots containing the experimental data and the fitted function.

To run the Perl scripts navigate the Terminal (Mac/Linux/Unix computers) or the Command Prompt (Cmd, Windows computers) to one of the folders and write (for example using the blue branch data inside the folder `green_blue`):

```
perl fit_blue.pl
```

and press the RETURN key.

You will see the iterations from gnuplot's fit function in the Terminal/Cmd and the generation of a new log file `fit.log` for this fit. The related `pdf` file, here `graph_blue_log.pdf` (log-log plot) or `graph_blue_lin.pdf` (linear plot) will be overwritten.

To generate a logarithmic plot uncomment (i.e. remove the # signs) inside the Perl

script in front of the lines:

```
# set log x
# set log y
```

Also, change the line:

```
set output 'graph_blue_lin.pdf'
```

to

```
set output 'graph_blue_log.pdf'
```

or to some other file name for the logarithmic plots.

To create linear plots comment out the lines `set log x` and `set log y`, i.e., setting a # sign in front of these lines, change the file name of the graph by the `'set output'` command and re-run the Perl script.

References

1. Wyszecki G, Stiles WS. Color Science. Second edition. Wiley New York; 1982.
