# Supplementary material for: Homeostasis at different backgrounds: The roles of overlayed feedback structures in vertebrate photoadaptation
Source: PLoS One. 2023 Apr 28;18(4):e0281490. doi: 10.1371/journal.pone.0281490 (PMC10146485; doi:10.1371/journal.pone.0281490)
Supplement: Attachment — Submitted filename: rebuttal_letter_revision_PONE-D-23-01979.pdf [file pone.0281490.s009.pdf]

Changes made in the revised manuscript based on the comments by Reviewer #1

Changes made in the manuscript with respect to the comments by Reviewer #1 are indicated in red in the marked-up copy of the revised manuscript.

Reviewer #1: In line with their previous works on homeostatic controllers, Ruoff and colleagues address here the behaviour of basic controllers with respect to different but constant backgrounds. They first carefully analyse the dynamics of 8 motifs in response to a step perturbation at different backgrounds and show that these motifs can be divided into 2 classes, depending on their resetting timing, which is shown to be independent on weather they are inflow or outflow controllers. The dynamics of selected motifs is illustrated in the main paper while other motifs are described in the supplementary material. This gives a comprehensive overview of the response of the different motifs to perturbations (in terms of amplitude and speed, but also in relation to Weber law, which states that the perception of a "just noticeable" difference between a reference state and a slightly higher state is proportional to the reference state).

In the second part of the paper the authors focus on the photoreceptor system in vertebrates. This system contains 3 overlayed feedback loops whose the molecular components are well described. They involve calcium and cGMP, as well as the CNG channel and NCKX pump. After an estimation of the parameter values based on experimental observations, the authors study the adaptation of the system to pulse and to step perturbations. For the pulse perturbation, the authors found that the threshold (light stimulus needed to get a response of a given amplitude) - background relationship follows a Weber law for large background and a Stephen power law for small background, in agreement with the experimental observations. For the step perturbation, they found that the amplitude of the cGMP excursion decreases monotonically with the increase of the background (as expected) but that the response time first decreases and then increases with the background. This latter, non-intuitive prediction is related to observations done on turtle photoreceptors. Finally, the authors analyse in detail the role of the feedback loops. They found that 2 antagonistic feedbacks allow a robust adaptation while a third, negative feedback impairs the robustness of the adaptation but contributes to keep calcium level low (thus preventing high cytotoxic levels).

The paper is well structured and clearly written. The authors provide an elegant way to understand the kinetics of the vertebrate photoreceptor system and made clear links with experimental observations. The analysis of the various motifs provide a solid basis to understand the "logic" of the photoreceptor system but possibly also other systems. The codes to reproduce each figure are made available.

Response: *We would like to thank the reviewer for the positive assessment.*

Reviewer #1: "I was just wondering if other models exist for the photoreceptor system in vertebrates and how they differ from the present one, regarding the molecular components taken into account and/or the kinetics."

Response: *Yes, there is an extensive literature on modeling retinal photoadaptation. We agree that a comparison of our 'homeostatic model' with previous calculations is in place. We have therefore added near the end (just before the 'Conclusion and outlook' section) the section 'Other model approaches'. It gives an overview of previous model calculations. Since none of the earlier calculations/models used a 'homeostatic approach', we added, at the end of this section the following paragraph:*
"Our approach, although primarily kinetic in nature, differs from previous adaptation

models by looking at photoadaptation from a robust homeostatic viewpoint. In this respect we agree with Billman [57] that homeostatic approaches are still underappreciated and are far too often ignored as a central organizing principle in physiology."

Changes made in the revised manuscript based on the comments by Reviewer #2

Changes made in the manuscript with respect to the comments by Reviewer #2 are indicated in blue in the marked-up copy of the revised manuscript.

Reviewer #2: "In the article entitled "Homeostasis at different backgrounds: the roles of overlayed feedback structures in vertebrate photoadaptation" by Grini et al., the authors classify the resetting behavior of eight possible basic integral regulatory motifs into two classes and discuss how the multiple feedback loops in molecular models of vertebrate photoadaptation are involved in controlling homeostasis. The work is original, has not been reported elsewhere, and the methods and analysis are of a high standard. The description is also adequate. However, some parts of the paper are confusing and there are several points that should be revised."

Response: *Thank you for your positive assessment and for your comments on how the manuscript can be improved. We have revised the manuscript at several places according to your comments, and describe the made changes in more detail below.*

Reviewer #2: "First, this reviewer's main concern is that this article should be divided into two separate studies, the study on the classification of basic integral controller motifs, and vertebrate photoadaptation molecular model dynamics. It is one outcome to discuss the existence of multiple feedback loops in the molecular model of vertebrate photoadaptation, each of which shows similarities to integral controller motifs, and their molecular dynamics. On the other hand, the finding that the possible basic integral controller motifs can be divided into two classes is also an achievement. Each of these is an independent achievement, but combining them into a single paper has blurred the point of contention. While it is possible to publish as is by addressing the following concerns, this reviewer believes that one option is to separate the independent results and publish them as two concise papers."

Response: *Although splitting this manuscript into two separate parts may be an option, we do not wish to do so. The findings on the controller motifs are not independent of our proposed 3-feedback light adaptation model. They provide the actual basis for it. We further feel that a splitting into two separate manuscripts will lead to unnecessary fragmentation of our work, which we want to avoid. However, as a response to the Academic Editor's comment, we have now explicitly described that the paper comes in two parts: one related to the analysis of the basic controller motifs, and another one, where retinal photoreceptor responses are analyzed on basis of the controller motifs responses, which leads to the 3-feedback light adaptation model. Please, see our changes made in response to the comment by the Academic Editor.*

Reviewer #2: "The reviewer was difficult to understand the meaning and significance of psychophysical laws (Weber's law and Stevens' power law). For example, there was a statement "Weber's law is followed when a "just noticeable difference" (threshold) of 1% of the controlled variable's set-point was considered." in the main text. What is the significance of following Weber's law? Similarly, the reviewer could not understand the significance of being able to express this in terms of Stevens' power law. These points

could be explained in more detail for the unfamiliar reader."

Response: *We feel it is of interest that the eight controller motifs follow Weber's and Stephens' law, since these laws are also found in experimental adaptation studies (see f eks. part IV in the textbook by Kandel et al, Ref. 21). In order to make the psychophysical laws more accessible to the unfamiliar reader, we have included in the section 'Psychophysical laws' a brief introduction. In addition, references are included such that the reader can get a more concise view.*

Minor comments by Reviewer #2:

• "p.2, Line 47: The constant C is not present in Eq 2."

Response: *C is now included.*

• "p.2, Line 47: Isn't w the reference wight? Why the sudden change to general stimulus here."

Response: *yes, in the example w was weight, but can in fact be any other stimulus. We have now included the sentence:* **"Instead of weight, w can generally be any other stimulus."**

• "p.3, Line 83: It should describe what the black arrow represents."

Response: *We have now included the sentence:* **"Black arrows indicate synthesis and removal of the controller variable E."**

• "p.5, Line 140: Which one is Fig 4a panel (c) referring to? Perhaps there should be a comma after Fig 4a?"

Response: *We have now included a comma.*

• "p.7, Line 170: The description of (Fig 1) after m2 and m8 is desirable."

Response: *We have now included '(Fig 1)' after m2 and m8.*

• "p.8, Line 193: Major points have been made, but at least a brief mention should be made of Figs 8c and 8d."

Response: *Yes, we agree. We have now added the sentence:* **"Figs 8c and d show how ΔA_{max} and t_{max} depend on the background k_4, respectively."**

• "p.8, Line 194: The reviewer believes this subsection should be moved to supporting information S3 Text, including Fig 9 because it is a little off the main subject."

Response: *Antithetic control is an alternative kinetic mechanism to ensure integral control within a negative feedback loop to ensure robust homeostasis. The concept of antithetic control was developed by M. Khammash's group in Basel. Since we use antithetic control later in the photoadaptation model (Fig 13) to describe the transport of Ca^{2+} and K^+ out of the cell by potassium-dependent sodium-calcium exchangers (NCKX), we wish to emphasize this control mode explicitly and therefore have the scheme in Fig 9 in the main manuscript. There is also the point we make that antithetic and zero-order controllers behave dynamically identical as outlined in the main paper, but shown in detail in S3 Text. To motivate having the antithetic control scheme in the manuscript, we have changed the first sentence in section 'Controller m2 with antithetic integral control' to:*

"Since we later will use bimolecular (antithetic) control [14,19] to describe the simultaneous removal of Ca^{2+} and K^+ out of a photoreceptor cell by potassium-dependent sodium-calcium exchangers (NCKX), we illustrate here how scheme m2 works with antithetic integral control (Fig 9)."

• "p.9, Line 205: Eq 12, not Eq 11?"

Response: *Please note that Eq 12 is Eq 11, but now under zero-order conditions. This defines A_{set}, as*

outlined in Eq. 12. The reference to Eq 12 on line 205 (line 225 in marked-up copy) is correct.

• "p.9, Line 222: This part should also be briefly explained at least for Fig 11 c and 11d."
Response: *We have now added the sentence:* **"Figs 11c and d show how ΔA_{max} and t_{max} depend on the background k_3, respectively."**

• "p.11, Line 247: CNG channels are not familiar to the general reader. Appropriate references should be cited."
Response: *With respect to CNG channels we have now cited the textbook by Marks et al. and the research paper by Hsu and Molday. The latter work is later used to extract rate parameters for the photoadaptation model.*

• "p.11, Line 249: No hyphen is required. Generally, "phosphodiesterase" is used."
Response: *We have corrected it.*

• "p.12, Eq 21: This notation should correspond to the notation with vleak in Fig 13."
Response: *We have now written v_{leak} in Eq 21 as in Fig 13, and at two other places.*

• "p.13, Line 310: Eq 11 wrong?"
Response: *Reference to Eq 12 is correct, since this is the zero-order version (with respect to E) of Eq 11.*

• "p.13, Lines 307-312: It is interesting ... possibly toxic Ca2+ concentration. I feel that these sentences are off the main line. Is it really necessary?"
Response: *We feel this is indeed an interesting aspect to discuss. As robust perfect adaptation could be considered to be an evolutionary goal by itself, we have here the situation where the Ca inhibition of the CNG channel 'destroys' the robust perfect adaptation of cGMP (induced by the two other Ca-related feedback loops). The role of Ca inhibition of the CNG channel is apparently to avoid too high cytosolic Ca levels. It is well documented that too high Ca levels lead to apoptosis, i.e. to cell death. To substantiate this aspect, we have slightly rewritten the last sentence of this section and cited the work by He et al. on Ca-induced apoptosis in photoreceptor cells:*
"However, such an antithetic control of cGMP without CNG channel inhibition by Ca_i^{2+} would lead to high Ca_i^{2+} concentrations and thereby to possible apoptosis of photoreceptor cells [46].

• "Fig 14 and Fig 14 Legends: What is the unit of measure for the vertical axis in Fig 14b?"
Response: *The experimental data we used were presented by their respective authors as normalized data, either in % or on the scale from 0-1. In order to make this fact more clear, we added the word 'Normalized' in the beginning of the legend of Fig 14.*

• "p.13, Line 314: Where did "experimental values" come from? Please cite the exact reference."
Response: *We have now cited work (Refs 32, 34, 47, 48), where cGMP and Ca_i^{2+} concentrations have been estimated.*

• "p.16, Lines 378-379: "Instead of ... Ca2+ and K+ [16]." Does this sentence make sense? Shouldn't the extra information in the middle be removed because it makes it difficult to understand the main points?"
Response: *The original papers on antithetic control (Ref 14, 15) considered uncatalyzed systems. We have recently considered catalyzed antithetic control (Ref 16). Eqs 21 and 22 write the antithetic control of cGMP by Ca^{2+} in an uncatalyzed transport of Ca^{2+} and K^+ out of the cell. Since the NCKX transporter actually catalyzes the transport, we simply wanted to say that one could have also used an catalyzed antithetic controller as described in Ref 16 on general grounds. We agree that the sentence was difficult to*

grasp. We have now restated the sentence to:
"Instead of using the term k(Ca_i^{2+})(K^+), one could have explicitly included the NCKX transporter protein, as generally outlined in [16] for catalyzed antithetic controllers.

Changes made in the revised manuscript based on the comments by Reviewer #3

Changes made in the manuscript with respect to the comments by Reviewer #3 are indicated in orange in the marked-up copy of the revised manuscript.

• "The research here described is exclusively computational. No mention was made on any statistical evaluation of the results, but I do not know if this kind of simulation allows any since it is based on LSODA. a linear Solver for Ordinary Differential Equations. I do not feel adequate to judge if it is necessary."
Response: *We have not used LSODA, but LSODE, which is the acronym of 'Livermore Solver of Ordinary Differential Equations'. LSODA is a variant of LSODE, and there are many more variants of LSODE. Considering the reviewer's comment, we have slightly changed the first sentence in the section 'Calculations and parameter estimations' to:*
"Computations were performed by using LSODE [26], which is part of a set of Fortran solvers at the Lawrence Livermore National Laboratory (https://computing.llnl.gov/projects/odepack)."

Changes made in the revised manuscript based on the comments by the Academic Editor

Changes made in the manuscript with respect to the comments by Academic Editor are indicated in violet in the marked-up copy of the revised manuscript.

• "It is fundamental to clarify both biological and computational aspects of this study (as highlighted by the reviewers) to make this work suitable for publication. I therefore suggested major revision."
Response: *We have made it more clear that the paper consists of two parts. Starting line 21 we have added the following sections:*
"In this paper we explore the influence of background perturbations on a set of eight basic negative feedback (controller) motifs [11]. We found that some of the motifs show an astonishing analogy to retinal photoreceptor adaptation when various background illuminations are applied.
The paper consists of two major parts. In the first part results from a systematic study of all eight controller motifs are shown. In the second part we show how certain of these controller motifs can provide an understanding about the kinetics of retinal photoreceptor adaptation. All eight feedback motifs show robust perfect homeostasis due to the implementation of integral control."

The Results and discussion section is now divided into 'Analyses of controller motifs' (line 111) and 'Implications to photoreceptor adaptation' (line 245).
