# Supplementary material for: Homeostasis at different backgrounds: The roles of overlayed feedback structures in vertebrate photoadaptation
Source: PLoS One. 2023 Apr 28;18(4):e0281490. doi: 10.1371/journal.pone.0281490 (PMC10146485; doi:10.1371/journal.pone.0281490)
Supplement: Attachment — Submitted filename: changes made in 2nd rev.pdf [file pone.0281490.s010.pdf]

Changes made in the revised manuscript based on the comments by Reviewer #1

Changes made in the manuscript with respect to the comments by Reviewer #1 are indicated in red in the marked-up copy of the revised manuscript.

Reviewer #1: In line with their previous works on homeostatic controllers, Ruoff and colleagues address here the behaviour of basic controllers with respect to different but constant backgrounds. They first carefully analyse the dynamics of 8 motifs in response to a step perturbation at different backgrounds and show that these motifs can be divided into 2 classes, depending on their resetting timing, which is shown to be independent on weather they are inflow or outflow controllers. The dynamics of selected motifs is illustrated in the main paper while other motifs are described in the supplementary material. This gives a comprehensive overview of the response of the different motifs to perturbations (in terms of amplitude and speed, but also in relation to Weber law, which states that the perception of a "just noticeable" difference between a reference state and a slightly higher state is proportional to the reference state).

In the second part of the paper the authors focus on the photoreceptor system in vertebrates. This system contains 3 overlayed feedback loops whose the molecular components are well described. They involve calcium and cGMP, as well as the CNG channel and NCKX pump. After an estimation of the parameter values based on experimental observations, the authors study the adaptation of the system to pulse and to step perturbations. For the pulse perturbation, the authors found that the threshold (light stimulus needed to get a response of a given amplitude) - background relationship follows a Weber law for large background and a Stephen power law for small background, in agreement with the experimental observations. For the step perturbation, they found that the amplitude of the cGMP excursion decreases monotonically with the increase of the background (as expected) but that the response time first decreases and then increases with the background. This latter, non-intuitive prediction is related to observations done on turtle photoreceptors. Finally, the authors analyse in detail the role of the feedback loops. They found that 2 antagonistic feedbacks allow a robust adaptation while a third, negative feedback impairs the robustness of the adaptation but contributes to keep calcium level low (thus preventing high cytotoxic levels).

The paper is well structured and clearly written. The authors provide an elegant way to understand the kinetics of the vertebrate photoreceptor system and made clear links with experimental observations. The analysis of the various motifs provide a solid basis to understand the "logic" of the photoreceptor system but possibly also other systems. The codes to reproduce each figure are made available.

Response: *We would like to thank the reviewer for the positive assessment.*

Reviewer #1: "I was just wondering if other models exist for the photoreceptor system in vertebrates and how they differ from the present one, regarding the molecular components taken into account and/or the kinetics."

Response: *Yes, there is an extensive literature on modeling retinal photoadaptation. We agree that a comparison of our 'homeostatic model' with previous calculations is in place. We have therefore added near the end (just before the 'Conclusion and outlook' section, starting line 485) the section 'Other model approaches'. It gives an overview of previous model calculations. Since none of the earlier calculations/models used a 'homeostatic approach', we added, at the end of this section the following paragraph:*

"Our approach, although primarily kinetic in nature, differs from previous adaptation models by looking at photoadaptation from a robust homeostatic viewpoint. In this respect we agree with Billman [63] that homeostatic approaches are still underappreciated and are far too often ignored as a central organizing principle in physiology."

Changes made in the revised manuscript based on the comments by Reviewer #2

Changes made in the manuscript with respect to the comments by Reviewer #2 are indicated in blue in the marked-up copy of the revised manuscript.

Reviewer #2: "In the article entitled "Homeostasis at different backgrounds: the roles of overlayed feedback structures in vertebrate photoadaptation" by Grini et al., the authors classify the resetting behavior of eight possible basic integral regulatory motifs into two classes and discuss how the multiple feedback loops in molecular models of vertebrate photoadaptation are involved in controlling homeostasis. The work is original, has not been reported elsewhere, and the methods and analysis are of a high standard. The description is also adequate. However, some parts of the paper are confusing and there are several points that should be revised."

Response: *Thank you for your positive assessment and for your comments on how the manuscript can be improved. We have revised the manuscript at several places according to your comments, and describe the made changes in more detail below.*

Reviewer #2: "First, this reviewer's main concern is that this article should be divided into two separate studies, the study on the classification of basic integral controller motifs, and vertebrate photoadaptation molecular model dynamics. It is one outcome to discuss the existence of multiple feedback loops in the molecular model of vertebrate photoadaptation, each of which shows similarities to integral controller motifs, and their molecular dynamics. On the other hand, the finding that the possible basic integral controller motifs can be divided into two classes is also an achievement. Each of these is an independent achievement, but combining them into a single paper has blurred the point of contention. While it is possible to publish as is by addressing the following concerns, this reviewer believes that one option is to separate the independent results and publish them as two concise papers."

Response: *Although splitting this manuscript into two separate parts may be an option, we do not wish to do so. The findings on the controller motifs are not independent of our proposed 3-feedback light adaptation model. They provide the actual basis for it. We further feel that a splitting into two separate manuscripts will lead to unnecessary fragmentation of our work, which we want to avoid. However, as a response to the Academic Editor's comment, we have now explicitly described that the paper comes in two parts: one related to the analysis of the basic controller motifs, and another one, where retinal photoreceptor responses are analyzed on basis of the controller motifs responses, which leads to the 3-feedback light adaptation model. Please, see our changes made in response to the comment by the Academic Editor.*

Reviewer #2: "The reviewer was difficult to understand the meaning and significance of psychophysical laws (Weber's law and Stevens' power law). For example, there was a statement "Weber's law is followed when a "just noticeable difference" (threshold) of 1% of the controlled variable's set-point was considered." in the main text. What is the significance of following Weber's law? Similarly, the reviewer could not

understand the significance of being able to express this in terms of Stevens' power law. These points could be explained in more detail for the unfamiliar reader."

Response: *We feel it is of interest that the eight controller motifs follow Weber's and Stephens' law, since these laws are also found in experimental adaptation studies (see f eks. part IV in the textbook by Kandel et al, Ref. 21). In order to make the psychophysical laws more accessible to the unfamiliar reader, we have included in the section 'Psychophysical laws' a brief introduction (starting line 41). In addition, references are included such that the reader can get a more concise view.*

Minor comments by Reviewer #2:

- "p.2, Line 47: The constant C is not present in Eq 2."
Response: *C is now included.*

- "p.2, Line 47: Isn't w the reference wight? Why the sudden change to general stimulus here."
Response: *yes, in the example w was weight, but can in fact be any other stimulus. We have now included the sentence (starting line 60):* **"Instead of weight, w can generally be any other stimulus."**

- "p.3, Line 83: It should describe what the black arrow represents."
Response: *We have now included the sentence (line 97):* **"Black arrows indicate synthesis and removal of the controller variable E."**

- "p.5, Line 140: Which one is Fig 4a panel (c) referring to? Perhaps there should be a comma after Fig 4a?"
Response: *We have now included a comma, see line 159.*

- "p.7, Line 170: The description of (Fig 1) after m2 and m8 is desirable."
Response: *We have now included '(Fig 1)' after m2 and m8; see line 189.*

- "p.8, Line 193: Major points have been made, but at least a brief mention should be made of Figs 8c and 8d."
Response: *Yes, we agree. We have now added the sentence (starting line 212):* **"Figs 8c and d show how ΔA_{max} and t_{max} depend on the background k_4, respectively."**

- "p.8, Line 194: The reviewer believes this subsection should be moved to supporting information S3 Text, including Fig 9 because it is a little off the main subject."
Response: *Antithetic control is an alternative kinetic mechanism to ensure integral control within a negative feedback loop to ensure robust homeostasis. The concept of antithetic control was developed by M. Khammash's group in Basel. Since we use antithetic control later in the photoadaptation model (Fig 13) to describe the transport of Ca^{2+} and K^{+} out of the cell by potassium-dependent sodium-calcium exchangers (NCKX), we wish to emphasize this control mode explicitly and therefore have the scheme in Fig 9 in the main manuscript. There is also the point we make that antithetic and zero-order controllers behave dynamically identical as outlined in the main paper, but shown in detail in S3 Text. To motivate having the antithetic control scheme in the manuscript, we have changed the first sentence in section 'Controller m2 with antithetic integral control' to (starting line 215):*
"Since we later will use bimolecular (antithetic) control [14,19] to describe the simultaneous removal of Ca^{2+} and K^{+} out of a photoreceptor cell by potassium-dependent sodium-calcium exchangers (NCKX), we illustrate here how scheme m2 works with antithetic integral control (Fig 9)."

- "p.9, Line 205: Eq 12, not Eq 11?"

Response: *Please note that Eq 12 is Eq 11, but now under zero-order conditions. This defines A_{set}, as outlined in Eq. 12. The reference to Eq 12 on line 205 (line 226 in marked-up copy) is correct.*

• "p.9, Line 222: This part should also be briefly explained at least for Fig 11 c and 11d."
Response: *We have now added the sentence (line 244):* **"Figs 11c and d show how ΔA_{max} and t_{max} depend on the background k_3, respectively."**

• "p.11, Line 247: CNG channels are not familiar to the general reader. Appropriate references should be cited."
Response: *With respect to CNG channels we have now cited appropriate references (Refs 37-39; see line 278).*

• "p.11, Line 249: No hyphen is required. Generally, "phosphodiesterase" is used."
Response: *We have corrected it (line 282).*

• "p.12, Eq 21: This notation should correspond to the notation with vleak in Fig 13."
Response: *We have now written v_{leak} in Eq 21 as in Fig 13, and at two other places.*

• "p.13, Line 310: Eq 11 wrong?"
Response: *Reference to Eq 12 is correct, since this is the zero-order version (with respect to E) of Eq 11.*

• "p.13, Lines 307-312: It is interesting ... possibly toxic Ca2+ concentration. I feel that these sentences are off the main line. Is it really necessary?"
Response: *We feel this is indeed an interesting aspect to discuss. As robust perfect adaptation could be considered to be an evolutionary goal by itself, we have here the situation where the Ca inhibition of the CNG channel 'destroys' the robust perfect adaptation of cGMP (induced by the two other Ca-related feedback loops). The role of Ca inhibition of the CNG channel is apparently to avoid too high cytosolic Ca levels. It is well documented that too high Ca levels lead to apoptosis, i.e. to cell death. To substantiate this aspect, we have slightly rewritten the last sentence of this section and cited the work by He et al. on Ca-induced apoptosis in photoreceptor cells (starting line 343):*
"However, such an antithetic control of cGMP without CNG channel inhibition by Ca_i^{2+} would lead to high Ca_i^{2+} concentrations and thereby to possible apoptosis of photoreceptor cells [49].

• "Fig 14 and Fig 14 Legends: What is the unit of measure for the vertical axis in Fig 14b?"
Response: *The experimental data we used were presented by their respective authors as normalized data, either in % or on the scale from 0-1. In order to make this fact more clear, we added the word 'Normalized' in the beginning of the legend of Fig 14.*

• "p.13, Line 314: Where did "experimental values" come from? Please cite the exact reference."
Response: *We have now cited the work (Refs 35, 37, 50, 51), where cGMP and Ca_i^{2+} concentrations have been estimated (line 347).*

• "p.16, Lines 378-379: "Instead of ... Ca2+ and K+ [16]." Does this sentence make sense? Shouldn't the extra information in the middle be removed because it makes it difficult to understand the main points?"
Response: *The original papers on antithetic control (Ref 14, 15) considered uncatalyzed systems. We have recently considered catalyzed antithetic control (Ref 16). Eqs 21 and 22 write the antithetic control of cGMP by Ca^{2+} in an uncatalyzed transport of Ca^{2+} and K^+ out of the cell. Since the NCKX transporter actually catalyzes the transport, we simply wanted to say that one could have also used an catalyzed antithetic controller as described in Ref 16 on general grounds. We agree that the sentence was difficult to*

grasp. We have now restated the sentence to (starting line 411):
"Instead of using the term k(Ca_i^{2+})(K^+), one could have explicitly included the NCKX transporter protein, as generally outlined in [16] for catalyzed antithetic controllers.

Changes made in the revised manuscript based on the comments by Reviewer #3

Changes made in the manuscript with respect to the comments by Reviewer #3 are indicated in orange in the marked-up copy of the revised manuscript.

• "The research here described is exclusively computational. No mention was made on any statistical evaluation of the results, but I do not know if this kind of simulation allows any since it is based on LSODA. a linear Solver for Ordinary Differential Equations. I do not feel adequate to judge if it is necessary."
Response: *We have not used LSODA, but LSODE, which is the acronym of 'Livermore Solver of Ordinary Differential Equations'. LSODA is a variant of LSODE, and there are many more variants of LSODE. Considering the reviewer's comment, we have slightly changed the first sentence in the section 'Calculations and parameter estimations' to (starting line 74):*
"Computations were performed by using LSODE [26], which is part of a set of Fortran solvers at the Lawrence Livermore National Laboratory (https://computing.llnl.gov/projects/odepack)."

Responses to the comments by Reviewer #3 which were in file 'revisione.docx'

• "The manuscript entitled 'Homeostasis at different backgrounds: The roles of overlayed feedback structures in vertebrates photoadaptation' by Jonas V. Grini, Melissa Nygård and Peter Ruoff describes the mathematical modeling of a quite complex control circuit regulating photoadaptation in vertebrates. Although some simplifications were necessary in order to build the model, the authors nicely demonstrate the significance of each of three feedback loops that guarantee the resilience and the adaptability of the system.
Though the manuscript is a little complicated to read in certain parts, the complexity of the model was clearly explained and completely illustrated. Some adjustments could be made to help the readers follow the simulations and understand the impact of the work."
Response: *Thank you for your positive assessment and comments.*

• "The Introduction paragraph should illustrate if previous attempts at modeling the vertebrate photoadaptation process have ever been undertaken. Moreover, a more thorough description of the biological background would be needed for the reader to understand the meaning of its formalization."
Response: *In response to the same comment by Reviewer #1, i.e. to give an overview of previous modeling approaches, we have added an overview, which is described in the section "Other model approaches" at the end of the manuscript (starting line 485). We wish to keep the organization of our manuscript by first showing the responses of negative feedback loops at different backgrounds (which was our starting point) and then show the example of the influence of background illumination on photoreceptor cell responses as a biological example. This arrangement is the reason why the overview of other model approaches on retinal photoreceptor adaptation comes at the end of the manuscript.*

To give an overview of the biological background we have added the following paragraph in the section "Implications to photoreceptor adaptation" (starting line 249):
"In mammals and other animals photoadaptation occurs mainly in the retina, which consists of five basic classes of neurons: photoreceptors, bipolar cells, ganglion cells, horizontal cells, and amacrine cells, where each of them come in different subclasses. These neurons are arranged in layers and form a complex interaction network [21,32,33]. Our focus here is on the light-sensitive photoreceptor cells, which according to their physical shape are characterized as rods and cones, and differ in their sensitivity to light. Rods and cones occur in all retinas with the exception of the skate [33]."

• "As the authors mention at the end of the results section, the model was set with parameters from very different organisms. When choosing this path, the authors should illustrate what data are available and if the photoadaptation could actually follow a similar model in the different organisms considered. That is, do turtles control photoadaptation in the same way of monkeys? When this model of photoadaptation actually evolved? Are the actors in this pathway conserved among vertebrates? Are the Ca2+, K+, cGMP concentrations even similar in all vertebrates?"
Response: *The model in Fig 13 is considered to be conserved among vertebrate organisms (including mammals and amphibians). We have therefore emphasized the word 'vertebrate' in the figure legend (outlined in orange color). Available experimental data on the molecular components we found in the literature are outlined in Fig 14. As described in the text they are based on salamander and bovine rods. There are few experimental data on free cellular cGMP concentrations in rods, but the values for mammals and amphibians are relatively close, i.e. in the order of 4 μM (Ref 51) and 6 μM (Ref 35), respectively. The same appears also to be the case for free cytosolic Ca concentration, which we referred to be in the range between 300-500 nM according to Ref 37. We are unsure what the reviewer means by: 'When this model of photoadaptation actually evolved?'.*

• "Is the classification of homeostatic controllers in two classes a novelty? The authors should provide previous literature excursus on this topic, in order to highlight the contribution of this work to the field."
Response: *In 'Feedback motifs investigated' (lines 93-94) we refer to previous work (Ref 11), where it is shown that the set of eight basic controller motifs (Fig 1) divide into two classes, i.e. into inflow and outflow controllers. This is not novel. However, this work now shows (this is the novel finding) that when considering background perturbations the motifs divide, according to their response kinetics, into two classes, but now based on whether the compensatory flux is of the activation or derepression type. These two types of compensatory fluxes are outlined in Fig 1 in respectively brown and green color. We also describe explicitly that the controllers response kinetics split into two classes independent whether they are inflow or outflow controllers. Please, see description in section 'Analyses of controller motifs' in revised ms, lines 123-132.*

• "The authors remark that the three feedback loops do not allow a robust perfect adaptation of cGMP. Would this have a consequence in adaptation of the system to a following stimulus? That is, would the perception be lowered to the same stimulus when administered after the first time? This could have a role in the adaptation to a continuous condition of light or dark."
Response: *Yes, in the case of the three overlayed feedback loops, perfect robust adaptation in cGMP is lost, which has a consequence on the adaptation of cGMP with respect to perturbations which add or remove cGMP. This is illustrated in Fig 19d. If one takes cGMP concentration in phase 1 as a reference, you see that cGMP concentration is increased in phase 2 when the perturbation adds cGMP, but is lower than the reference when cGMP is removed, as indicated in phase 3. Note, that the applied perturbations in Fig 19 are steps, i.e. an applied continuous condition.*

• "Fig. 1. Dashed lines are not described in the legend."

Response: *We have described the meaning of the dashed lines in the main text (starting line 98), just before Fig 1:*
"Dashed lines represent signaling events which lead to the activation (plus signs) or inhibition (minus signs) of target reactions."

• "Lines 121-122. Please explain the impact that this assumption could have to the results of the simulation, with the respect to the adherence to the biological process. Is it negligible?"
Response: *As indicated in text the assumption that activations are first order with respect to the concentration of the activation species is a simplification. The assumption of activation cannot be neglected, but neglects possible saturation and controller breakdown at high activator concentrations. We now state explicitly that the assumption we make neglects that and cite the work of Ang and McMillen, who first showed possible saturation effects and controller breakdown (starting line 141):*
"This assumption neglects possible saturation and controller breakdown at high activator concentrations [31]."

• "In some Figure legends the kinetics is referred to as zero-order Michaelis-Menten. This is a very specific kinetic bound to enzymology and lead readers with biological background to think that the model is depicting an enzymatic process. Is it? If ion fluxes are also taken into consideration, the authors would prefer being more generic and talk about Hill sigmoid functions. Moreover, the authors claim that E degradation is a zero-order Michaelis-Menten. If I understand correctly, this would mean that the system is always saturated with E≫k7, which would make k7 useless by definition. Is this the case? Finally, k3 is not described in Figure 3 and 7, and k4 is not described in Figure 5"
Response: *Yes, we think in terms of enzyme and transporter catalyzed processes. The assumption of zero-order Michaelis-Menten kinetics is one of several requirements which can lead to integral control and robust perfect adaptation. It assumes that an enzyme/transporter which removes or transports E is always saturated with E. It is an idealized assumption. Cooperativity (Hill sigmoid functions) are not obligatory, but can be included (see for example Ref 29). Low k_7 is not useless by definition! Even if k_7 is very low, it needs to be there to avoid negative concentrations in E. k_7 also reflects the accuracy of the controller (as lower k_7 as more accurate the controller resets to the controller's set-point). Please, see Refs 11,13 for details. Other requirements for integral control are antithetic mechanisms (Refs 14-16), or autocatalysis in E combined with first-order removal of E (Refs 17-19).*
We have now included a description of k_3 and k_4 in respectively Figs 3 and 7, and in Fig 5 (outlined in orange color).

• "Figure 4, 6, 8, 11 and 15, 16 legends are very complex to read. The values reported would be better in a Table associated to the figure, or even better another graph where effect on E0 is shown, since this is the likely cause for the time for resetting variation. This would also better illustrate the concepts in lines 144-146, 166-167."
Response: *We have considered to include additional tables in relation to Figs 4, 6, 8, 11, 15, and 16. We decided against it, as we felt that all relevant data should be associated within these figures and not distributed across the manuscript. In the Supporting Information S1 Programs we have provided python scripts, where the interested reader can test the behavior of the controllers in detail using the data provided in the legends of Figs 4, 6, 8, 11, 15, and 16.*

• "In the supporting information 'S3 Text', the legend of Fig. S2 does not describe any subpanel. The orange line is very difficult to see."
Response: *We have now increased the line thicknesses in Figs S2a and S2b, and included a description of all three panels in the figure legend.*

• "Line 241. Why despite? Ca2+ levels are tightly regulated exactly because it is a versatile cellular signal... please rephrase"

Response: *Seems we look slightly differently at this. 'Versatile' means 'flexible', 'all-round', 'many-sided', etc. Despite that Ca is a flexible signal, it is also tightly controlled, which we feel restricts Ca versatile behavior. Anyway, we have reworded the sentence to (line 272):*

"While Ca^{2+} is a versatile cellular signal its levels are also tightly regulated [38]."

• "Fig. 13 legend. 'the removal of cyclic guanosine monophosphate (cGMP) by phospho-diesterases (PDE), via transducin and the activation of PDE by internal Ca2+ (Ca2+i)', are these two different paths? They are not clearly identifiable in the figure, please refer to the different color in the legend. Does Ca2+ activation apply to both? 'GC is inhibited/derepressed by Ca2+' is confusing."

Response: *The rate equations show that the two different colors refer to the light induced removal of cGMP. One (rate constant k_4, outlined in blue) is a background, while the other (k_2, outlined in red) is a light perturbation on top of the background. They are components of the same path. To make this more clear, we have added the following sentence to the figure legend:*

"In the figure this path is split into two components, one background with rate constant k_4 (outlined in blue), and a perturbation on top of the background (rate constant k_2, outlined in red)."

Concerning the terms 'inhibition/derepression' it is calcium which (dependent on the perturbation) controls the inflow of cGMP either by increasing the inhibition of cGMP synthesis or by decreasing it (the latter is termed derepression). We have slightly changed the sentence to:

"GC is inhibited/derepressed by Ca_i^{2+} to keep cGMP under homeostatic control."

• "Lines 248-250. It is not clear if Ca2+ activates both reactions of cGMP removal, as stated in eq. 20. Please explain better the background data"

Response: *Yes, Ca activates the light dependent removal of cGMP, which includes k_2 and k_4. This should be now more clear as we have added (according to the question by the reviewer above) the following sentence in the legend of Fig 13:*

"In the figure this path is split into two components, one background with rate constant k_4 (outlined in blue), and a perturbation on top of the background (rate constant k_2, outlined in red)."

In addition, we added (line 281) the following:

"....removal of cGMP (with rate constants k_2 and k_4)...."

• "Figure 13. K11,n should be on the dashed arrow from cGMP. K6 should come from the extracellular medium, I guess?"

Yes, this is correct. We have changed Fig 13 in this respect.

• "Line 293. 'K+ synthesis' is not an adequate term: K+ cannot be synthesized, it can only enter from the outside. Please rephrase."

Response: *Correct, we have changed 'synthesis' to 'inflow' (line 325 in marked-up copy).*

• "S7 should be S6"

Correct, we have changed S7 to S6

• "Fig. 15. Are K+ concentrations in physiological range?"

Response: *No, they are far too low since we have not considered to describe the sodium ion and potassium ion currents in detail. However, since the structure of the Ca outflow by NCKX is analogous to an antithetic controller, we have used a simplified antithetic control scheme which includes potassium ion. To address this point, we have slightly reworded our previous statement that sodium ion was not considered*

to the following (line 338 in marked-up copy):
"Apart from that, we have not considered sodium ion and potassium ion currents."

Changes made in the revised manuscript based on the comments by the Academic Editor

Changes made in the manuscript with respect to the comments by Academic Editor are indicated in violet in the marked-up copy of the revised manuscript.

• "It is fundamental to clarify both biological and computational aspects of this study (as highlighted by the reviewers) to make this work suitable for publication. I therefore suggested major revision."
Response: *We have made it more clear that the paper consists of two parts. Starting line 21 we have added the following sections:*
"In this paper we explore the influence of background perturbations on a set of eight basic negative feedback (controller) motifs [11]. We found that some of the motifs show an astonishing analogy to retinal photoreceptor adaptation when various background illuminations are applied.
The paper consists of two major parts. In the first part results from a systematic study of all eight controller motifs are shown. In the second part we show how certain of these controller motifs can provide an understanding about the kinetics of retinal photoreceptor adaptation. All eight feedback motifs show robust perfect homeostasis due to the implementation of integral control."

The Results and discussion section is now divided into 'Analyses of controller motifs' (line 111) and 'Implications to photoreceptor adaptation' (line 246).
