# Supplementary material for: Homeostasis at different backgrounds: The roles of overlayed feedback structures in vertebrate photoadaptation
Source: PLoS One. 2023 Apr 28;18(4):e0281490. doi: 10.1371/journal.pone.0281490 (PMC10146485; doi:10.1371/journal.pone.0281490)
Supplement: Attachment — Submitted filename: revisione.docx [file pone.0281490.s008.docx]

The manuscript entitled ‘Homeostasis at different backgrounds: The roles of overlayed feedback structures in

vertebrates photoadaptation’ by Jonas V. Grini, Melissa Nygård and Peter Ruoff describes the mathematical modeling of a quite complex control circuit regulating photoadaptation in vertebrates. Although some simplifications were necessary in order to build the model, the authors nicely demonstrate the significance of each of three feedback loops that guarantee the resilience and the adaptability of the system.

Though the manuscript is a little complicated to read in certain parts, the complexity of the model was clearly explained and completely illustrated. Some adjustments could be made to help the readers follow the simulations and understand the impact of the work.

Major remarks

- The Introduction paragraph should illustrate if previous attempts at modeling the vertebrate photoadaptation process have ever been undertaken. Moreover, a more thorough description of the biological background would be needed for the reader to understand the meaning of its formalization.
- As the authors mention at the end of the results section, the model was set with parameters from very different organisms. When choosing this path, the authors should illustrate what data are available and if the photoadaptation could actually follow a similar model in the different organisms considered. That is, do turtles control photoadaptation in the same way of monkeys? When this model of photoadaptation actually evolved? Are the actors in this pathway conserved among vertebrates? Are the Ca2+, K+, cGMP concentrations even similar in all vertebrates?
- Is the classification of homeostatic controllers in two classes a novelty? The authors should provide previous literature excursus on this topic, in order to highlight the contribution of this work to the field.
- The authors remark that the three feedback loops do not allow a robust perfect adaptation of cGMP. Would this have a consequence in adaptation of the system to a following stimulus? That is, would the perception be lowered to the same stimulus when administered after the first time? This could have a role in the adaptation to a continuous condition of light or dark.

Minor remarks.

- Fig. 1. Dashed lines are not described in the legend.
- Lines 121-122. Please explain the impact that this assumption could have to the results of the simulation, with the respect to the adherence to the biological process. Is it negligible?
- In some Figure legends the kinetics is referred to as zero-order Michaelis-Menten. This is a very specific kinetic bound to enzymology and lead readers with biological background to think that the model is depicting an enzymatic process. Is it? If ion fluxes are also taken into consideration, the authors would prefer being more generic and talk about Hill sigmoid functions. Moreover, the authors claim that E degradation is a zero-order Michaelis-Menten. If I understand correctly, this would mean that the system is always saturated with E>>>k7, which would make k7 useless by definition. Is this the case? Finally, k3 is not described in Figure 3 and 7, and k4 is not described in Figure 5
- Figure 4, 6, 8, 11 and 15, 16 legends are very complex to read. The values reported would be better in a Table associated to the figure, or even better another graph where effect on E0 is shown, since this is the likely cause for the time for resetting variation. This would also better illustrate the concepts in lines 144-146, 166-167.
- In the supporting information ’S3 Text’, the legend of Fig. S2 does not describe any subpanel. The orange line is very difficult to see.
- Line 241. Why despite? Ca2+ levels are tightly regulated exactly because it is a versatile cellular signal… please rephrase
- Fig. 13 legend. ‘the removal of cyclic guanosine monophosphate (cGMP) by phospho-diesterases (PDE), via transducin and the activation of PDE by internal Ca2+ (Ca2+i )’, are these two different paths? They are not clearly identifiable in the figure, please refer to the different color in the legend. Does Ca2+ activation apply to both? ‘GC is inhibited/derepressed by Ca2+’ is confusing.
- Lines 248-250. It is not clear if Ca2+ activates both reactions of cGMP removal, as stated in eq. 20. Please explain better the background data
- Figure 13. K11,n should be on the dashed arrow from cGMP. K6 should come from the extracellular medium, I guess?
- Line 293. ‘K+ synthesis’ is not an adequate term: K+ cannot be synthesized, it can only enter from the outside. Please rephrase.
- Line 339. S7 should be S6
- Fig. 15. Are K+ concentrations in physiological range?
